# Network analysis reveals rare disease signatures across multiple levels of biological organization

**DOI:** 10.1038/s41467-021-26674-1

**Published:** 2021-11-09

**Authors:** Pisanu Buphamalai, Tomislav Kokotovic, Vanja Nagy, Jörg Menche

**Affiliations:** 1 CeMM Research Center for Molecular Medicine of the Austrian Academy of Sciences, Lazarettgasse 14, AKH BT 25.3, 1090 Vienna, Austria; 2 Department of Structural and Computational Biology, Max Perutz Labs, University of Vienna, Campus Vienna BioCenter 5, 1030 Vienna, Austria; 3 Ludwig Boltzmann Institute for Rare and Undiagnosed Diseases, Lazarettgasse 14, AKH BT 25.3, 1090 Vienna, Austria; 4 Department of Neurology, Medical University of Vienna, Währinger Gürtel 18‐20, 1090 Vienna, Austria; 5 Faculty of Mathematics, University of Vienna, Oskar-Morgenstern-Platz 1, 1090 Vienna, Austria

**Keywords:** Computational models, Data integration, Genetic interaction, Regulatory networks, Genetics research

## Abstract

Rare genetic diseases are typically caused by a single gene defect. Despite this clear causal relationship between genotype and phenotype, identifying the pathobiological mechanisms at various levels of biological organization remains a practical and conceptual challenge. Here, we introduce a network approach for evaluating the impact of rare gene defects across biological scales. We construct a multiplex network consisting of over 20 million gene relationships that are organized into 46 network layers spanning six major biological scales between genotype and phenotype. A comprehensive analysis of 3,771 rare diseases reveals distinct phenotypic modules within individual layers. These modules can be exploited to mechanistically dissect the impact of gene defects and accurately predict rare disease gene candidates. Our results show that the disease module formalism can be applied to rare diseases and generalized beyond physical interaction networks. These findings open up new venues to apply network-based tools for cross-scale data integration.

## Abstract (web summary)

Despite the clear causal relationship between genotype and phenotype in rare diseases, identifying the pathobiological mechanisms at various levels of biological organization remains a practical and conceptual challenge. Here, the authors introduce a network approach for evaluating the impact of rare gene defects across biological scales.

## Introduction

Over the past 2 decades, rapid advances in DNA sequencing technology allowed us to uncover the genetic basis of over 6000 rare diseases^1–3^. In contrast to common diseases, which are typically characterized by a complex interplay between multiple genetic and environmental factors, rare diseases can often be pinpointed to a single genetic lesion. Rare diseases thus offer unique opportunities to mechanistically dissect the relationship between genetic aberrations and their phenotypic consequences, which can then inform targeted treatment strategies. For individual rare diseases, this potential for a molecularly rooted, personalized medicine could already be demonstrated, for example in rare immunodeficiencies^4–6^, neurodevelopmental^7,8^, and metabolic disorders^9,10^. At the same time, the costs and extended timelines of these individual efforts also highlight the need for novel, systematic approaches for investigating the large number of rare diseases that still remain uncharacterized. To this end, several practical and conceptual challenges need to be overcome:

First, rare disease phenomena cover a wide spectrum, from highly cell-type or organ-specific phenotypes to heterogeneous, syndromic diseases that affect the whole body. Our understanding of how a genetic aberration impacts various scales of biological organization between genotype and clinical phenotype is very limited. Second, the enormous complexity within and between different organizational scales, such as the transcriptome, proteome, intra- or intercellular communication, also poses important technical challenges: How can we identify and integrate the most relevant data? Third, the rarity of many conditions with monogenic origins implies that data are usually scarce. Traditionally, rare diseases have been studied following a one-gene, one-pathway, one-disease paradigm. A systematic approach for transferring knowledge from one rare disease to another, and for investigating differences and commonalities between different diseases, is still missing.

In this work, we propose a network-based framework for systematically investigating rare diseases that addresses these challenges, and, in turn, use the large number of rare diseases with a well-described genetic origin to deepen our understanding of disease-associated perturbations of molecular networks. Specifically, we introduce a multiplex network approach for integrating different network layers that represent different scales of biological organization ranging from the genome to the transcriptome and the phenome. A systematic characterization of the network signatures of all rare diseases with known genetic causes allowed us to identify the connectivity patterns that determine the importance of a particular scale of biological organization for a given rare disease. Finally, we explored how these systems-level insights may help contextualize individual genetic lesions, investigate the impact of disease heterogeneity, and be translated into clinically actionable tools for the genetic diagnosis of rare disease patients with unknown gene defects.

## Results

### Constructing a gene network bridging molecular and phenotypic scales

Rare diseases affect many scales of biological organization which, conversely, may provide valuable information for elucidating a particular gene defect. At the genetic level, for example, interplay between genetic variants can modulate phenotypic outcomes^11^ or even completely rescue disease-associated variants^12^. At the protein level, members of the same complex or pathway are often implicated in similar phenotypes^13,14^ and expression patterns of a particular gene can reveal affected cell types and tissues^15–17^. Finally, phenotypic similarities with known human or animal model gene defects can guide the annotation of genetic variants with unknown consequences^18^.

**Fig. 1:**
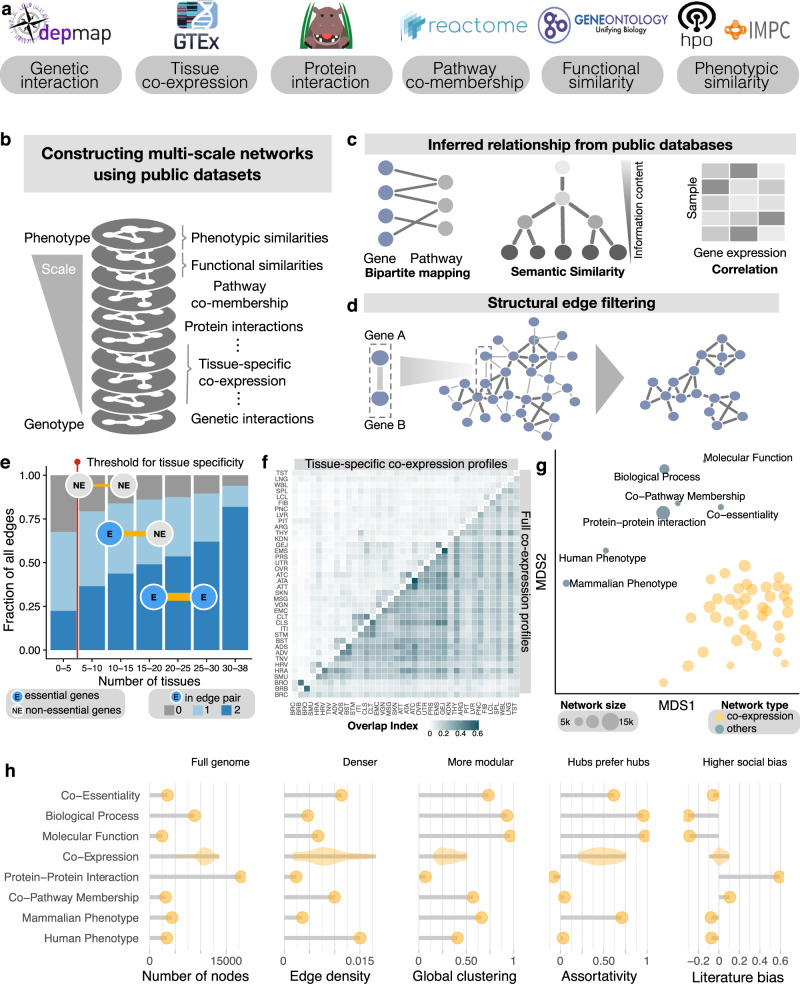
Construction and characterization of the cross-scale multiplex network. **a** Data resources for the major biological levels of organization represented in the multiplex network. **b** The multiplex network consists of 46 network layers, each representing a particular type of gene relationships, ranging from genetic interactions to phenotypic similarity. **c** Methods used for inferring networks: bipartite mapping was used to build gene relationships based on common annotations, e.g., pathways; semantic similarity was used to define relationships based on annotation similarity; correlation analyses were used to identify co-expression. **d** Weighted and dense networks were subsequently filtered based on structural network criteria for extracting the most relevant interactions. **e** Co-expressed gene pairs found in a higher number of tissues tend to be essential, reflecting core cellular functions. Edges found in five or fewer tissues were considered tissue-specific. **f** Full co-expression profiles are highly similar between tissues and thus redundant (lower triangle). The removal of core transcription profiles reveals tissue-specific patterns of the co-expression networks (upper triangle). **g** The multi-dimensional scaling (MDS) plot based on edge overlap similarity of all networks shows a clear distinction between major types and subtypes of the network layers. **h** Major network characteristics for all considered network layers: number of nodes edge density, global clustering, assortativity and social bias, as measured by the correlation between node degree and number of associated publications. The values of the 38 individual co-expression networks are shown in the form of a distribution.

To integrate these diverse relationships into a unifying, gene-centric framework, we constructed a multiplex network comprised of several layers: The nodes in each layer represent genes, the links represent their respective relationship at a particular scale of biological organization, ranging from direct interactions between gene products at the molecular level to phenotypic similarity of associated diseases at the phenotype level (Fig. 1a, b). We compiled information from seven databases and, where appropriate, applied a range of techniques for extracting gene relationship, such as bipartite mapping, ontology-based semantic similarity metrics and correlation-based relationship quantification, as well as filtering based on both statistical and network structural criteria^19^ (Fig. 1c, d and Supplementary Figs. 1 and 2, see Methods for details). The resulting multiplex network consisted of 46 layers containing over 20 million relationships between 20,354 genes (Supplementary Data 1 and 2). The relationships represent six major biological scales: (*i*) The genome scale, where links represent genetic interactions derived from CRISPR screening in 276 cancer cell lines^20^. (*ii*) The transcriptome scale, where interactions represent co-expression, i.e., co-variability of gene transcription levels indicative of higher-level regulatory mechanisms. We included both pan-tissue and tissue specific networks derived from RNA-seq data across 53 tissues in the GTEx database^17^. (*iii*) The proteome scale, where links represent physical interactions between gene products obtained from the HIPPIE database^21^. (*iv*) The pathway scale, where links represent pathway co-membership derived from the REACTOME database^22^. (*v*) The scale of biological processes and molecular functions, where links represent similar functional annotations derived from the Gene Ontology^23^. (*vi*) The phenotypic scale, where links represent similarity in annotated phenotypes derived from the Mammalian and Human Phenotype Ontologies (MPO and HPO)^24,25^.

### Characterizing the network architectures across biological scales

To characterize the resulting cross-scale gene relationships, we first quantified the global similarity between all pairs of network layers *A* and *B* by the overlap of their respective sets of edges *E*: ${S}_{{AB}}=|{E}_{A}\cap {E}_{B}|/{\min }( | {{E}}_{A}|,|{E}_{B} | )$. The highest similarities were found within the transcriptomic scale: co-expression networks of different tissues have an overlap of up to $S=0.49$ (between brain tissues), compared to an average similarity of $S=0.05$ between networks of other scales. A major contribution to this elevated similarity is given by a core of links that is preserved across multiple tissues. We found that the proportion of links that connect essential genes increases with the number of tissues in which a particular link is present (Fig. 1e). This suggests that the common core is related to essential housekeeping activities. To represent pan-tissue and tissue-specific interactions separately, we extracted broadly preserved co-expression edges and considered them as a separate core transcription network layer, consisting of 12,364 nodes and 1,062,924 edges (Supplementary Fig. 1c, d, Methods). We further combined redundant tissue types, resulting in a final set of 38 tissue-specific networks used in the downstream analyses (Fig. 1f, Methods, Supplementary Data 3). These tissue-specific networks still form a recognizable cluster within the multidimensional scaling (MDS) projection of the relative similarities between all networks (Fig. 1g). The differences between tissues, however, are comparable with differences to networks of other scales (median similarity among tissues: $S=0.043$; similarity to other scales: $S=0.018$). The clear separation between most network layers (median similarity $S=0.033$) indicates that each layer contains unique information (Supplementary Fig. 3b). At the same time, a comparison with randomized networks reveals that a significant amount of interactions are preserved across levels of organization (Supplementary Fig. 3c, d), as shown by a significant similarity for 96.5% of all network pairs (empirical *p*-value < 0.05, see Methods). Finally, we noticed that the relative position of all network layers in Fig. 1g suggests a representative role for the protein–protein interaction (PPI) layer, which is located in a central position and close to the layers that directly encode phenotypic similarities.

We next compared the networks at the different biological scales in terms of five structural characteristics: genome coverage, overall connectivity, clustering, assortativity and literature bias (Fig. 1h, Methods). The results revealed a wide structural diversity: The network layer with the highest genome coverage is the PPI scale, covering 17,944 proteins. This is due to the combination of a large number of literature curated small-scale experiments and several large-scale screening efforts. Such systematic, genome-wide measurements also underlie the high coverage of the transcriptomic layers (with a total number of *N* = 17,432 genes across all tissues, and an average number of 10,527 genes per tissue, Supplementary Fig. 1d, see Methods for the filtering processes). Our incomplete understanding of how these molecular interactions translate into biological processes, however, is indicated by the low coverages observed among the functional and phenotypic levels (*N* = 2407 and 3342 for the molecular function and HPO networks, respectively). The high connectivity and clustering among these functional layers, in turn, is the basis for their predictive power for transferring gene annotations within functional clusters^11,20^ (e.g., edge density = $1.13\times 1{0}^{-2}$ and clustering = 0.73 for the co-essentiality network). The PPI represents the sparsest network (edge density = $2.359\times 1{0}^{-3}$; average density across all layers = $7.76\times 1{0}^{-3}$), which, in part, reflects the incompleteness of currently available data^26^. Curiously, the PPI is the only network in our collection that exhibits a (modest) level of disassortativity ($r=-0.08$), i.e., a tendency of hubs to connect preferentially to low-degree nodes, a property that was previously suggested to be a universal feature of biological networks^27^. Disassortativity may arise when the neighbors of high-interest nodes are mapped out more extensively than the interaction partners of these neighbors. For the PPI, this is likely to be the case in network data curated from hypothesis-driven, small-scale experiments, but can also occur in unbiased large-scale efforts (Supplementary Fig. 3e, f, Methods). A further characterization of curated and unbiased subsets of the PPI (Supplementary Fig. 4a, b, Methods) revealed that the relatively high literature bias present in the PPI, as measured by the correlation between the degree of a protein and the number of associated publications (Spearman’s *ρ* = 0.59, Supplementary Fig. 4b), is largely driven by its curated subset, which represents 87% of the full PPI. This emphasizes that despite recent efforts towards unbiased, high-throughput protein–protein interaction screening, a large fraction of the currently available PPI network information still reflects the reductionist, hypothesis driven research paradigm where new knowledge preferentially accumulates around proteins with an already known important function. This literature bias is notably absent in all other network layers.

In summary, the structural diversity observed among the individual network layers reflects both organizational principles intrinsic to a particular biological scale, as well as technical or historical details pertaining to the curation process of the underlying database (Supplementary Fig. 4c–f). We expect that this diversity further corresponds to complementary pieces of information contained in the different biological scales, collectively increasing their potential to drive novel insights into the relationships between rare disease genes.

### Identifying cross-scale network signatures of rare diseases

To investigate the connectivity patterns among rare disease genes, we collected 3953 genes associated with 3771 rare disease terms from the Orphanet database, the largest rare disease ontology and resource for genetic associations (Supplementary Data 4). Collectively, rare diseases represent an extraordinarily rich resource of causative genetic aberrations and their phenotypic consequences. For individual rare diseases, however, the situation is the opposite: Over 3501 diseases in the Orphanet database (~93%) are associated with fewer than five genes. This represents a major challenge for systematic, comparative rare disease research in general, and for network-based approaches in particular: Network approaches are based on the fundamental observation that genes associated with the same disease are not scattered randomly in molecular networks, but aggregate in disease-specific neighborhoods or “disease modules”^26,28^. However, the incompleteness of currently available network maps sets a lower bound for the number of genes that can be recognized as a connected module. This minimal number was estimated to be around 20 for the PPI network^26^, so that individually, only few rare diseases have a sufficiently large number of associated genes.

**Fig. 2:**
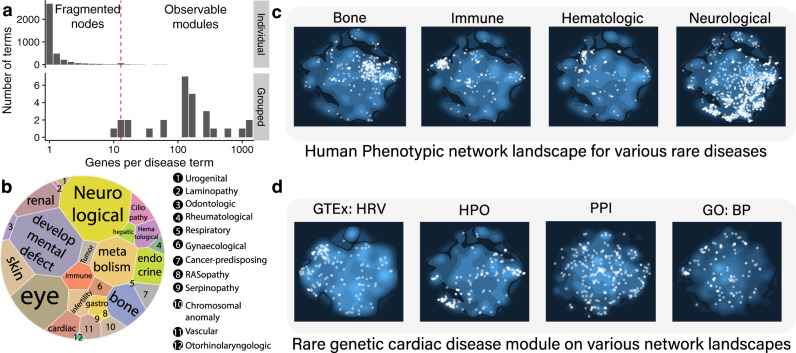
Rare disease grouping and network mapping reveal network- and disease-specific connectivity patterns. **a** Rare genetic diseases are typically associated with only a few genes and therefore remain fragmented on molecular networks. Grouping rare diseases by phenotypic similarity can overcome data scarcity and result in identifiable disease modules, thus allowing for further network-based inspection. **b** Voronoi treemap showing the 26 rare genetic disease groups used in this study. The size of each disease group is proportional to the number of associated genes. **c** Network landscape obtained using the node2vec embedding algorithm. Network distances between genes are preserved in the embedding and illustrate differential modularity of different rare disease groups on the Human phenotype similarity network layer. The bright dots represent disease associated genes and the blue contour map represents all genes in a network. **d** Localization of the rare cardiac disease group on different network layers.

We hypothesized that the disease module concept can be generalized to groups of rare diseases with closely related phenotypes. Collectively, these related rare diseases could thus reach the required minimum number of genes to form a recognizable disease module (Fig. 2a). To test this hypothesis, we used the hierarchical classification of rare diseases within the Orphanet Disease Ontology to aggregate rare diseases with similar phenotypes and collect all genes associated with their corresponding descendant terms. We identified a total of 26 rare genetic disease groups that are sufficiently broad or well-studied, respectively, to result in a number of associated genes required for network module approaches (i.e., more than 20), while retaining the pathophysiological specificity of rare disease phenotypes (Supplementary Fig. 5a). The disease groups range from smaller groups, such as RASopathy (ORPHA:536391) or rare genetic vascular diseases (ORPHA:233655) (with 20 and 22 associated genes, respectively), to large groups with over 1000 associated genes, such as rare genetic neurological disorder (ORPHA:71859) or rare developmental defect during embryogenesis (with 1649 and 1598 associated genes each). The average number of genes per disease group was 339 (Fig. 2b and Supplementary Data 5). Despite the wide range in the total number of associated genes per disease group, the average number of genes per disease term remains comparable across all disease groups, thus ensuring similar levels of disease specificity across the disease domain. In addition, there is only little overlap between the disease terms contained in the different groups, with 90.5% of all disease pairs being distinct (Jaccard Index < 0.1), indicating that the groups provide non-redundant disease definitions (Supplementary Fig. 5b).

We first inspected the network localization of the aggregated rare disease groups within two-dimensional network embeddings obtained from the node2vec algorithm^29^, which aims to preserve network distances between nodes (Supplementary Fig. 5c, Methods). Fig. 2c shows the resulting network landscape of the human phenotypic network with four rare disease groups highlighted: rare bone, immune, hematologic and neurological diseases (the complete landscape of all diseases in all networks can be explored via our MultiOmeExplorer web app: www.menchelab.com/MultiOmeExplorer, see also Supplementary Fig. 6). We found that all four disease groups localize within specific network neighborhoods. Given that the HPO network is based on phenotypic similarity of individual gene defects, this localization confirms that aggregating diseases based on disease ontology relationships indeed leads to groups of phenotypically related diseases. We further noticed that the different disease groups cover network areas of varying size, from highly localized immune diseases to more broadly spread neurological diseases. In part, this spread can be attributed to the larger number of genes associated with the latter disease group. More generally, it may reflect varying degrees of coherence and specificity among the phenotypic manifestations of the diseases represented within a particular group. The close relationship between the spread of disease-associated perturbations within molecular networks and the heterogeneity of clinical symptoms has previously been shown for complex diseases^30^. Similarly, the spread of the rare neurological disease cluster recapitulates the high level of comorbidities observed among affected patients. Finally, we noted that the proximity between neighborhoods is indicative of disease similarity, e.g., between rare immune and hematologic diseases, where the interplay between blood and immune system often leads to similar phenotypes.

We next inspected the network signatures of rare disease groups across different network layers. Figure 2d shows that rare genetic cardiac diseases are strongly localized on a heart-specific co-expression network (heart right ventricle; HRV) and the human phenotypic similarity network (HPO). The more dispersed signals on the PPI network and the network of shared biological processes (GO:BP), on the other hand, suggest that the respective genes might be involved in a broad range of molecular processes that cannot be adequately depicted in a two-dimensional projection.

### Quantifying network modularity of rare diseases

**Fig. 3:**
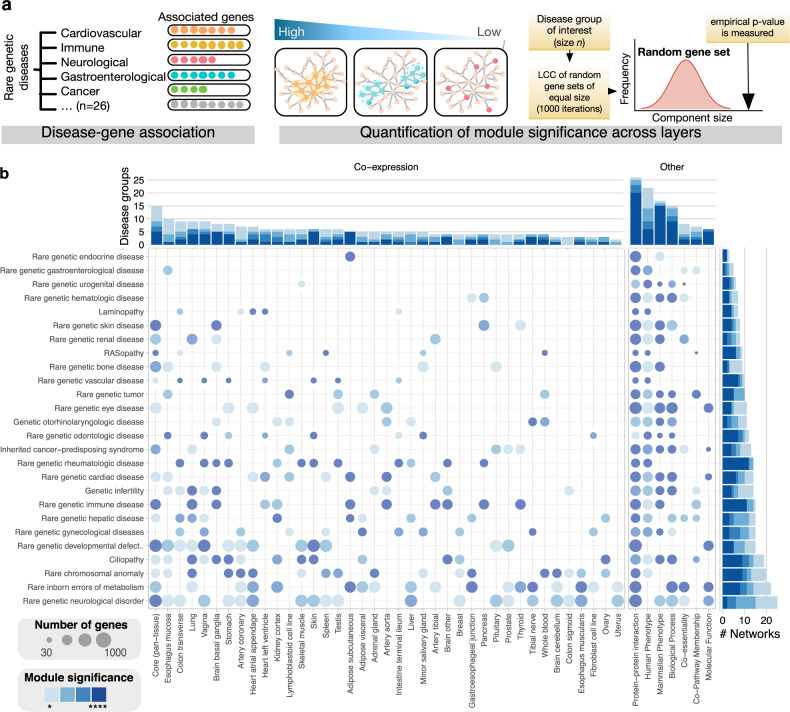
Multiplex network modularity of rare disease groups. **a** Pipeline for disease module significance assessment. The size of the largest connected component (LCC) for genes associated with rare genetic disease groups collected from Fig. 2 were used to determine network relevance. **b** The heatmap shows the modularity of all rare disease groups across all network layers as measured by the respective module size significance (*p* < 0.05: *, *p* < 0.01: **, *p* < 0.001: ***; *p* < 0.0001: ****, Benjamini–Hochberg corrected empirical *p*-values determined by node randomization, see Methods). In the tissue-specific network layers, only selected disease groups display pronounced modularity, often recapitulating known mechanisms and tissue specificities of particular rare diseases, but also revealing novel relationships. Network layers containing relationships that are relevant across biological levels of organization, such as protein–protein interaction, phenotypic and functional similarity networks, also display modularity across a wide range of disease groups.

The results so far indicate that the concept of disease modules, observed widely across complex diseases on PPI networks, can also be generalized to groups of rare diseases and to other network data representing relationships beyond the molecular scale of PPIs. Based on the heterogeneous degrees of modularity for different diseases and networks observed above, we further hypothesized that the degree of modularity can be related to the degree of relevance of the underlying information to a particular disease phenotype. To investigate this hypothesis and further dissect the characteristics of rare disease modules across biological scales, we systematically assessed all rare disease groups across all network layers. We quantified the level of modularity by the significance of the size of the largest connected component (LCC) of disease genes on a given network, as measured by the corresponding *z*-score compared to random gene sets (Fig. 3a, Methods). Figure 3b shows the module significance for all rare disease groups on all network layers summarized in one heatmap. We observed a high degree of differential modularity, i.e., the levels of localization vary greatly between disease groups and network layers. The largest number of significantly localized rare disease groups are found on the PPI network, the phenotypic networks (HP, MP), the core transcription network, and the network of shared biological processes. This consistent localization across a wide range of rare diseases confirms the existence of disease modules also for rare diseases. In contrast to the core transcription layer observed to be relevant across multiple disease groups, the tissue-specific co-expression networks provide a more disease-specific picture with unique signatures that reflect the molecular mechanisms that underlie a particular disease group on a given tissue. For example, the wider localization pattern of rare neurological disease genes in the phenotypic landscape observed in Fig. 2c corresponds to their significant modularity across co-expression networks in a wide range of tissues, which, in turn, reflects the often syndromic and heterogeneous phenotypes of these diseases.

### Using differential modularity to contextualize rare disease gene clusters

**Fig. 4:**
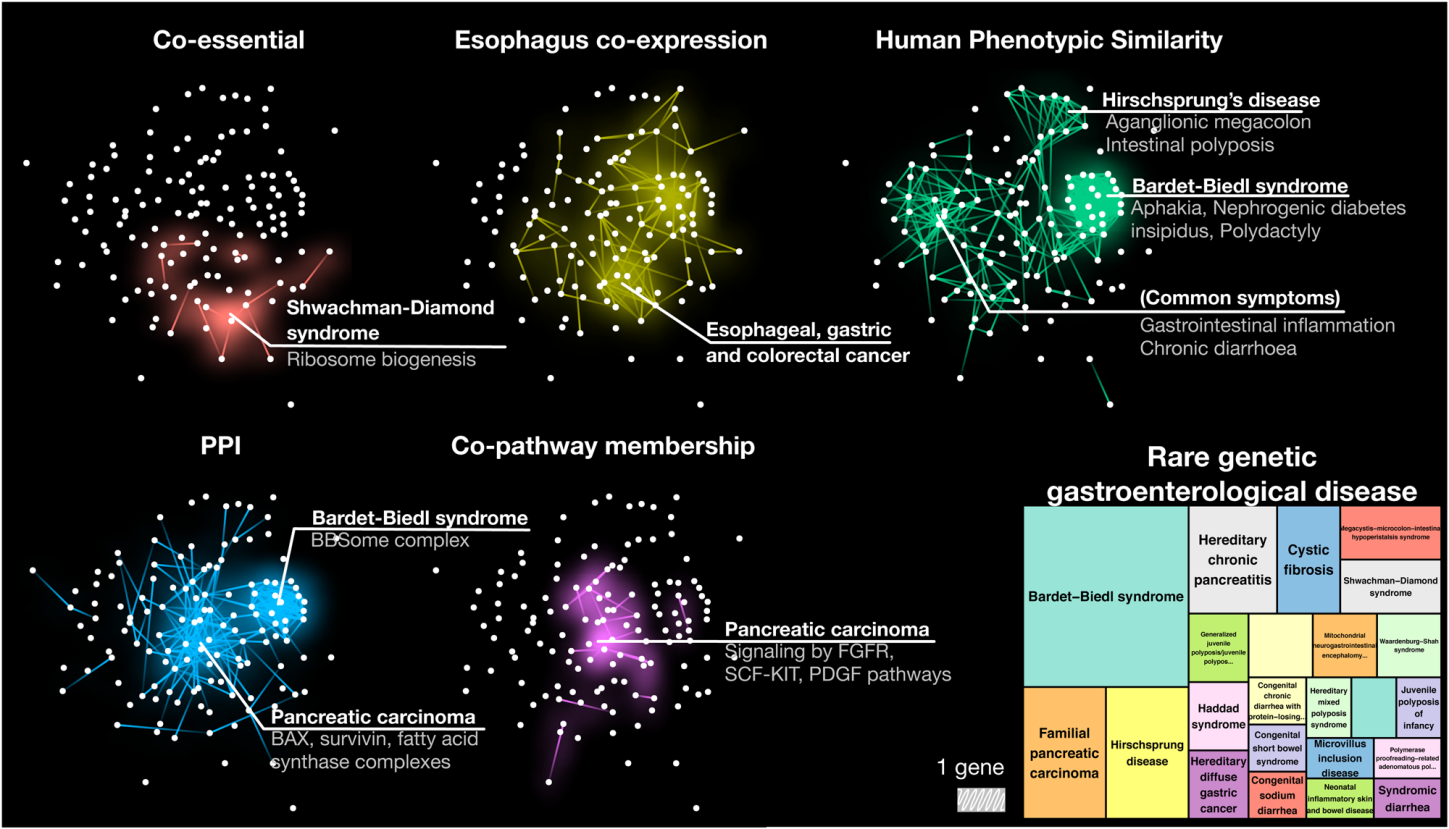
Network modules of rare genetic gastroenterological diseases across different levels of biological organization. Genes implicated in rare gastroenterological disease form significant modules on five network layers (compare with Fig. 3), which capture relevant relationships on different scales. Diseases in the disease group exhibit unique connectivity patterns in the network layers where their disease characteristics can be derived. For example, a strong phenotypic cluster of Bardet-Biedl syndrome genes can be derived from the protein complex (BBSome) whose defects lead to cilia dysfunction, while pancreatic carcinoma cluster is most observable on the pathway level where its causal genes interact physically and are also member of crucial signaling pathways. The treemap represents disease entries in Orphanet (leaf terms with at least two gene associations) that belong to the rare genetic gastroenterological disease group (69 diseases with only one gene association are not shown separately).

The individual layers within the cross-scale network capture different pathobiological mechanisms. The observed differential modularities can thus offer insights into the disease etiology specific to a particular layer. For example, rare genetic gastroenterological diseases, a disease group consisting of 92 disease terms with 140 associated genes, were found to be significantly localized on five network layers (Fig. 4). Detailed inspection and enrichment of these submodules (Supplementary Fig. 6a, b, Methods) enables us to interpret the disease characteristics within each layer: We found that genes causing the Bardet-Biedl syndrome (BBS) form pronounced clusters in the phenotypic and PPI layers. Together with the absence of modularity in other layers, this pinpoints that the emergence of this particular disease phenotype is mainly determined by interactions at the protein level, while co-essential, functional or pathway levels play less important roles. This observation is supported by our current knowledge of BBS pathological mechanisms: The proteins encoded by BBS genes form a complex crucial for transporting vesicles to cilia, a process whose defect is suspected to be a major cause of BBS^31^. At the same time, these proteins are of diverse functional character^32^ and involved in disparate pathways^33^, explaining the lower modularity on the respective network layers.

We also observed differential modularity among rare cancer-related disorders. Pancreatic carcinoma genes are involved in various apoptotic processes and significantly connected on both protein and pathway levels, highlighting the BAX and survivin protein complexes, and FGFR1, SCF-KIT, and PGDF signaling pathways, respectively. Modulation of gene expression involved in these processes has been reported to play a key role in pancreatic tumor growth^34,35^. Moreover, genes involved in cancers of gastrointestinal tracts (esophageal, gastric and colorectal cancer) form a strong cluster on the esophagus co-expression network, revealing their interconnected roles at the gene regulatory level.

Another notable cluster among rare gastroenterological diseases is given by the Shwachman-Diamond syndrome: 90% of all patients have a mutation in the SBDS gene, which is known to be involved in ribosome maturation, but otherwise poorly annotated. Recent studies indicate that the remaining 10% of patients with similar disease phenotypes have mutations in other genes involved in ribosome biogenesis^36,37^. The interactions between these genes and SBDS are only captured in the genetic (co-essentiality) and the transcriptomic (co-expression) layers.

These observations pave the way for a detailed mechanistic interpretation of how different network layers contribute to the etiology of individual rare diseases, as well as for identifying mechanisms that are shared among phenotypically related rare diseases.

### Modularity as quantification of pathobiological relevance

Our findings suggest that the significance of the network localization of rare disease genes on a particular layer of the cross-scale network may be used to quantify the pathobiological relevance of the respective level of biological organization for the disease. The information in Fig. 3b could thus be interpreted as a network-disease relevance score (π) for distinguishing information that truly reflects the system of interest from unrelated information and potential noise. Specifically, we hypothesized that if the associated genes of a disease group are significantly connected on a particular network, then the network has predictive power for discovering novel genes associated with the disease. In contrast, if the genes are scattered on a particular network, then it is likely uninformative for the discovery process. To test his hypothesis, we developed an informed multiplex network propagation algorithm, in which the overall probability *p*_*m*_ to visit a given layer *m* out of all *L* layers, ${p}_{m}={\sum }_{i=1}^{L}p({i|m})$, is proportional to its respective level of relevance *π*_*m*_, which can be achieved by incorporating the detailed balance condition ${\pi }_{m}p({m|n})={\pi }_{n}p({n|m})$ (Methods).

**Fig. 5:**
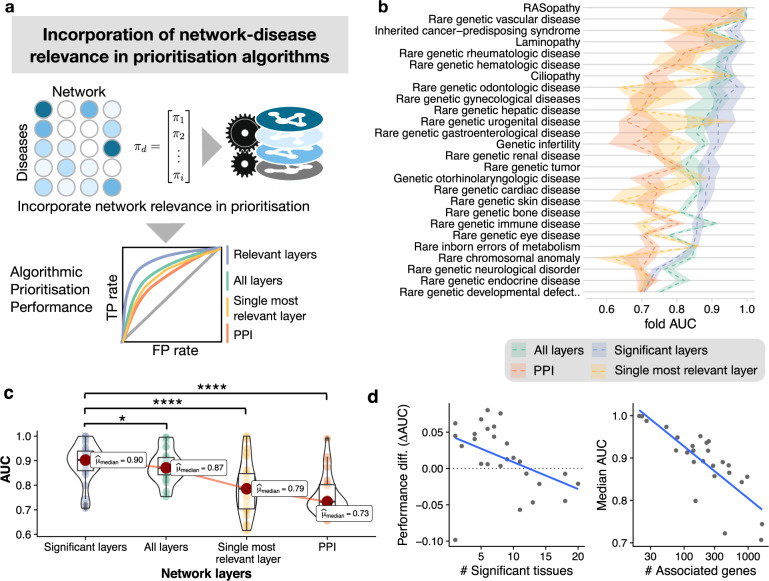
Using network modularity as relevance prior in the informed propagation algorithm for gene prioritization. **a** Schematic overview of the informed multiplex network propagation algorithm that incorporates modularity as measure of relevance of a particular network level for a given disease group. **b** Comparison of 10-fold cross-validation performance in rare disease gene retrieval for different choices of included networks: Informed algorithm with most relevant network (blue), all networks (green), the PPI (red), and the single most relevant layer for each disease (yellow). Dashed lines show median value across all folds, shaded areas represent the interquartile range. The retrieval performance indicates that disease mechanisms are generally better recapitulated by incorporating relevant networks only. **c** Comparison of the AUROC from all four methods. Utilizing the significant networks lead to more accurate disease gene retrieval compared to all networks, the single most relevant layer, or the PPI. (Bonferroni-Holm corrected Durbin-Conover test *p*-value = 0.026, 1.22e−6, and 3e-16 respectively). Threshold for *p*-values: *p* < 0.05:*, *p* < 0.01:**, *p* < 0.001:***, *p* < 0.0001:****; *n* = 26 rare disease groups across all network sets. Bounds of box represent 25th and 75th percentiles, center the median, whiskers 10th and 90th percentiles, respectively. **d** Factors correlated with the retrieval performance. The algorithm that incorporates all networks can outperform the informed algorithm for diseases with high levels of syndromicity, i.e., disease that manifest in multiple physiological systems (left, Spearman’s *ρ* = −0.53, corresponding *p*-value = 0.004). Decreasing functional relevance as the number of genes increases also led to lower predictive performance (right, Spearman’s *ρ* = −0.83, *p*-value = 1.94e-6). The corresponding *p*-value of correlation was determined by Fisher z-transformation, two-sided.

To validate the potential of this informed propagation algorithm for disease gene discovery, we performed a 10-fold cross-validation for the retrieval of associated genes for all disease groups and assessed the performance through the area under the receiver operating characteristic curve (AUROC, Fig. 5a). We compared four different scenarios incorporating (*i*) only the single most informative network (i.e., the one with the highest number of significantly localized disease groups; here: the PPI), (*ii*) the single most relevant network for each disease group (Supplementary Fig. 7a), (*iii*) all networks and (*iv*) only the most relevant networks (i.e., networks with a modularity significance of *p*-value < 0.05, Benjamini–Hochberg correction for multiple hypotheses). We found that all four different sets of networks performed reasonably well, with AUROC ranging from 0.65 to 0.95 (Fig. 5b), confirming the general applicability of multiplex network propagation to rare disease gene prediction. A comparison between the four methods revealed that incorporating only relevant network layers (median AUROC = 0.90) generally outperforms the PPI (median AUROC = 0.73), the most relevant single layer benchmark (median AUROC = 0.79), as well as the incorporation of all layers (median AUROC = 0.86), with corresponding Bonferroni–Holm corrected Durbin-Conover test *p*-value = 3e-16, 1.22e-6, and 0.002 respectively (Fig. 5c). We concluded that network modularity thus provides a network-based criterion to curate and integrate the most relevant data and levels of biological organization for a specific disease.

Interestingly, we also observed differences in retrieval performance related to characteristics of the diseases themselves: We found that syndromic disease groups, i.e., those with significant disease modules across multiple tissue types, tend to have lower retrieval performance and benefit from incorporating all tissue co-expression networks (Spearman’s *ρ* = −0.53, *p*-value = 0.004, Fig. 5d left panel, Supplementary Fig. 7b). On the one hand, this poses a challenge for disease groups that manifest various anatomical features such as rare genetic neurological disorders. On the other hand, this reflects limitations of broad ORDO disease group definitions such as rare genetic defects during embryological development. We further found that the retrieval performance correlated negatively with the number of genes associated with a particular disease group (Spearman’s *ρ* = −0.83, *p*-value = 1.94e-6, Fig. 5d right panel). These two factors are closely related, as both the syndromicity level and overall heterogeneity tend to increase as more genes are involved in the disease group. Taken together, these findings indicate that well defined disease groups with low to moderate number of associated disease genes are more likely to capture molecular disease characteristics at a level of specificity that results in better network-based predictions. This suggests that more fine-grained, mechanism-based disease definitions, together with high-resolution phenotyping will aid in further improving the predictive power of the introduced network methods.

To further dissect the contribution of individual networks and potential curation biases on the overall predictive power, we performed several additional benchmarks on different subsets of the multiplex network (Methods). Our comparisons between curated, unbiased and size-matched random subsets of the PPI indicate that the performance is largely driven by network size rather than potential literature biases in the interaction curation process (Supplementary Fig. 7c). We also evaluated the differences in performance upon removing individual layers, as well as groups of layers from the full multiplex network (Supplementary Fig. 7d). The results suggest that the performance is not driven by individual network layers and that the predictive power of the multiplex network can be best understood as a collective characteristic of all disease relevant layers.

### Application to candidate gene prioritization in rare disease patients

**Fig. 6:**
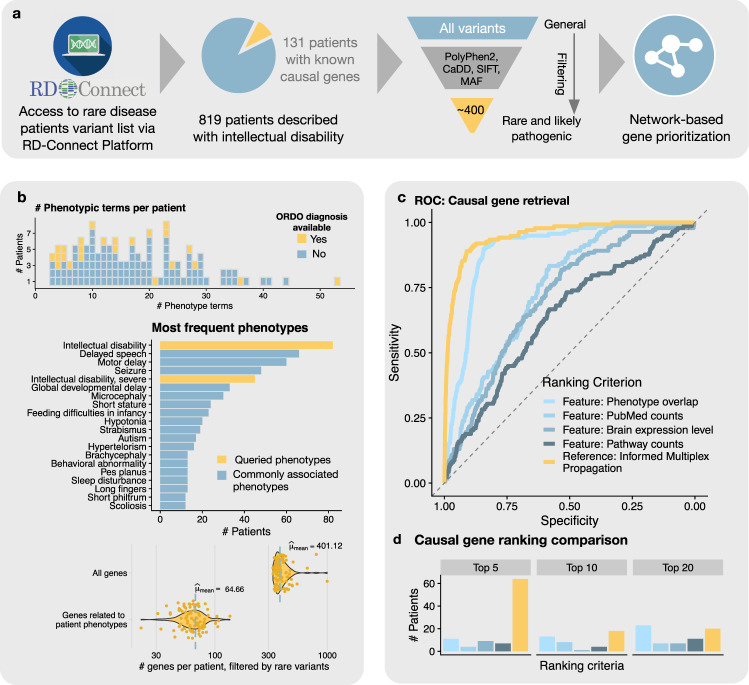
Patient cohort and gene prioritization performance. **a** Data access and filtering: Querying for intellectual disability phenotypes resulted in 819 patients, 131 of which were solved cases with rare and pathogenic variants in an average of over 400 genes. **b** Basic characteristics of patient variants, associated phenotypes and diagnoses. **c** ROC curves for the performance of causal gene prioritization of our approach (yellow, AUROC = = 0.95) and various gene level based benchmarks (AUROC between 0.59 and 0.87). **d** Number of patients for which the true causal gene was prioritized among the top five, 10, and 20 for all considered methods. The informed multiplex propagation placed the true causal gene among the top five ranked genes for 64 out of 131 patients (48.9%). For the purely gene-based methods, the causal gene was among the top five in only between 4 and 11 patients (3.1–8.4%).

Based on the performance of the informed multiplex propagation for retrieving genes across all rare disease groups we hypothesized that the method can also act as an additional evaluation metric for prioritizing genomic variants in individual rare disease patients. Starting point in a diagnostic setting is next-generation sequencing of a patient’s genome, typically yielding rare genomic variants (allele frequency < 1%) in dozens to hundreds of different coding regions, and with unknown consequences. These variants may be further filtered down, for example based on frequency in the general population, deleteriousness scoring, or segregation analysis, resulting in up to a few dozen high confidence candidate genes^38,39^. Identifying the one causal gene among them remains a critical challenge both in research and in clinical practice (Fig. 6a).

We tailored the informed propagation algorithm to individual patients by using seed genes associated with patient-specific phenotypes, combined with the network relevance scores from the corresponding Orphanet disease group (Supplementary Fig. 7e). Altogether, this enables us to perform patient-specific multiplex network propagation to prioritize candidate genes. We applied the method to filtered lists of genes with rare variants obtained from a cohort of 139 rare disease patients suffering from various neurological symptoms with intellectual disability as a predominant phenotype (Fig. 6b, Supplementary Fig. 7f, g, Supplementary Data 7, and Methods for details of the cohort). The causal variants of all patients were already confirmed and could thus be utilized for benchmarking. After standard methods of filtering for high confidence variants were exhausted, up to 997 candidate genes per patient remained (mean = 401.2). We found that our algorithm prioritizes causal genes with an overall AUROC of 0.95 (Fig. 6c). Furthermore, we benchmarked the performance of our method against predictions based on various gene-level properties, using the same data as in the network construction, specifically (1) pathway information, (2) expression level information, (3) literature counts and (4) phenotypic similarity (see Methods for details). Among these methods, phenotypic similarity was most predictive (AUC = 0.87), followed by literature counts (AUC = 0.72), expression information (AUC = 0.71) and pathway counts (AUC = 0.59). However, the informed multiplex propagation outperformed all gene-based methods (*p*-value = 8.39e-5, DeLong’s test of ROCs between our approach and the best performing gene-level method, i.e., phenotypic similarity).

Note that for the specific use case of gene variant prioritization in rare disease patients, it is instructive to not only consider the global ROC-based performance, but also the exact ranking of the causal gene when assessing the utility of the framework in research and clinical settings. In our cohort, the multiplex propagation method placed the true causal gene among the top five ranked genes for 64 out of 131 patients (48.9%). For the purely gene-based methods, the causal gene was among the top five in only between 4 and 11 patients (3.1–8.4%, Fig. 6d).

We also performed an additional benchmark using a temporal-holdout setting to ensure that the performance of our method is not primarily driven by confirmatory biases (Supplementary Fig. 8a, Methods). To this end, we curated a set of 21 patients with causal genes that were unknown at the time of network construction, thus minimizing the likelihood that their disease association contributed to information in any of the curated databases (Supplementary Fig. 8a, Methods). We found that the overall performance as measured by the AUROC remained high for all tested prediction methods. The observed slight reductions were within the 10-fold interquartile range in most cases and may also be attributed to the smaller sample size. For example, the informed multiplex propagation AUROC was reduced from 0.90 to 0.86 (Supplementary Fig. 8b). A closer inspection of the ranking showed that our framework maintained its proportion of true causal genes being ranked in the top five gene list, whereas almost all gene-based approaches had difficulties in retrieving them at highly ranked positions (Supplementary Fig. 8c, d).

## Discussion

In the context of complex diseases, numerous network-based studies have revealed an intimate relationship between genetic disease associations, their interaction patterns, and pathophysiological manifestations^40,41^. Most importantly, it was found that disease genes are not scattered randomly in molecular networks, but instead agglomerate in disease-specific modules^26^. Molecular networks can thus serve as maps to guide the search for new disease genes^42–44^, suggest drug repurposing^45–47^ and combination strategies^48,49^, or elucidate disease relationships^26,50^, to name but a few important applications.

Our work expands this concept in several directions: First, we showed that by aggregating individual gene defects into groups of related phenotypes, we can apply tools originally developed for common, polygenic diseases also to rare, monogenic diseases. Our comprehensive analysis of over 3,584 individual gene defects revealed that as a group, they exhibit network signatures similar to those observed for complex diseases. This opens up a wide range of network medicine tools and concepts to be applied to rare diseases. Existing tools, for example for prioritizing rare variant genes, often augmented by additional clinical data^51–55^, demonstrate the potential for network-based methods in this area.

We further showed that the central network medicine concept of disease modules can be generalized towards multiplex networks representing various levels of biological organization. Previous work relied prominently on physical protein–protein interactions, which have been mapped out systematically for nearly two decades^13,14,56,57^. Our analysis of 46 network layers containing over 20 million interactions showed that disease modules can be identified across a wide range of relevant gene relationships. We further found that the degree of modularity is indicative of the impact of disease-associated perturbations on a particular level of biological organization, and thereby determines the disease relevance of datasets from the respective level. The performance of the informed propagation algorithm for rare disease gene prediction demonstrates the practical utility of this finding. We expect that the general principle for identifying the most relevant datasets will be applicable in other contexts as well, including studies on cancer and other complex diseases. Indeed, as biomedical research is becoming more data intensive in general, and more biological network maps become available in particular^58,59^, new strategies for integrating diverse data are required. A range of methodologies have been developed for this purpose, including network-based strategies^60–63^ and advanced machine-learning approaches^64–66^. Our results could potentially enhance these strategies by using disease modularity as a criterion for curating and excluding potentially uninformative datasets.

Finally, to enable a broad community of researchers in the areas of rare disease, network medicine or biomedical data integration to build on our work, all datasets and algorithms presented in this work are publicly available.

## Methods

### Resources and network construction

Resources used in the multiplex network construction are listed in Table 1. We incorporated seven major databases, each representing distinct biological layers.

**Table 1:** Resources used for the multiplex network construction.

Layer representation	source
Biological process	GO (BP) release 2018-11-24, retrieved from http://purl.obolibrary.org/obo/go/go-basic.obo
Molecular function	GO (MF) release 2018-11-24, retrieved from http://purl.obolibrary.org/obo/go/go-basic.obo
Human phenotype	HPO release 2018-10-09, retrieved from http://purl.obolibrary.org/obo/hp.obo
Mammalian phenotype	MPO release 2018-11-23, retrieved from http://purl.obolibrary.org/obo/mp.obo
Co-essentiality	co-essential networks inferred from correlated fitness profile across diverse cancer cell lines from Kim et al.^20^.
Protein-protein interaction	HIPPIE v2.2 (release 2019-02-14), retrieved from http://cbdm-01.zdv.uni-mainz.de/mschaefer/hippie/download.php
Co-pathway membership	Reactome Pathways Gene Set, retrieved on 30 January 2019 from https://reactome.org/download/current/ReactomePathways.gmt.zip
Tissue co-expression	RNA-seq from GTEx v7, retrieved from the Expression Atlas https://www.ebi.ac.uk/gxa/experiments/E-MTAB-5214/

#### Protein–protein Interactions

Protein–protein interaction data was taken from the HIPPIE database^21^ and filtered for interactions with supporting PubMed articles. To assess the impact of interactions collected from small-scale, hypothesis-driven experiments compared to those stemming from large-scale, unbiased screens, we further collected the most recent versions of the two largest systematic high-throughput PPI studies: the Human Reference Interactome (HuRI) based on yeast two-hybrid (Y2H) screening^13^ (retrieved from http://www.interactome-atlas.org/data/HuRI.tsv on 31 May 2021), and the BioPlex interactome constructed from affinity-purification mass spectrometry profiling^67^ (retrieved from https://bioplex.hms.harvard.edu/data/BioPlex_293T_Network_10K_Dec_2019.tsv, on 31 May 2021). The PPI network layer can therefore be split into two categories: the unbiased PPI for interactions that are contained in any of these two resources, and the curated PPI for the remaining edges (Supplementary Fig. 4a).

#### Tissue Co-expression networks

Transcriptomic data is one of the most abundant publicly available high-throughput data. Differential expression profiles across tissues and cell types have been widely analyzed as a probe for disease specificity. In the context of network analyses, expression data has been used in two major ways: as a means to filter out genes from generic interactomes based on their expression level in a particular context of interest^13,60^, and for constructing co-expression networks. Here, we follow the latter approach, and use co-expression as a proxy for tissue- or cell type-specific functional and regulatory relationships. As primary resource we used the Genotype-Tissue Expression (GTEx) data^68,69^, which provides genome-scale expression profiles across 53 human tissues that have been used previously to construct co-expression networks^15,16^. We used the following pipeline:

1. We downloaded the GTEx expression profiles in the format of transcripts per million (TPMs) from the Expression Atlas (https://www.ebi.ac.uk/gxa/experiments/E-MTAB-5214/). The data was subsequently processed using the bioconductor package SummarizedExperiment (https://bioconductor.org/packages/release/bioc/html/SummarizedExperiment.html).
2. Tissues with a number of samples less than a minimum quality threshold similar to the GTEx Portal (*n* ≥ 70) were removed. These included fallopian tube (*n* = 14), ectocervix (*n* = 12), endocervix (*n* = 10) and urinary bladder (*n* = 24).
3. For the remaining 49 tissues, we further merged tissues with similar expression profiles to reduce redundancy and increase signals^70^. Most notably, brain regions (13 tissues) were merged into three major groups and relabeled by their anatomical entities (Supplementary Fig. 1a). This process not only merged potentially redundant tissues, but also increased sample sizes for some tissue groups that would otherwise have been undersampled. The resulting 38 tissue groups along with the sample sizes are shown in Supplementary Data 3.
4. The GTEx database contains an average of 29,779 ± 1972 transcripts per tissue. We next filtered for protein coding transcripts (e.g., exclusion of pseudogenes, long non-coding (lnc) RNAs, miRNAs, and other non-coding biotypes) by discarding transcripts without corresponding accession numbers in the UniProt Knowledgebase (www.uniprot.org) according to a query of MyGene (https://mygene.info, retrieved on 21 August 2019). Supplementary Data 10 lists the 21,310 transcripts in consideration, resulting in 17,716 ± 369 protein-coding genes per tissue (Supplementary Fig. 1b).
5. For each tissue, Spearman’s correlation coefficient (*ρ*) of all protein-coding gene pairs was used to determine the strength of their respective co-expression levels. Gene pairs with |*ρ*| ≤ 0.75 were discarded, resulting in 11,161 ± 1082 genes per tissue.
6. We applied a disparity filter^19^ to remove weak, structurally redundant edges and to extract the backbone of each network. Edges with a corresponding disparity filter *p*-value < 0.05 were selected. This process yielded 10,526 ± 1825 genes per tissue. Note that even though the number of nodes decreased only slightly, the disparity filter excluded a large amount of spurious correlations (median number of interactions before and after = 1.83e6 and 4.78e5, respectively, Supplementary Fig. 1b (right)). The disparity filter represents a dynamic cutoff, where lowly expressed genes tend to be removed and highly expressed genes tend to remain, while also allowing for the detection of lowly expressed genes that are strongly correlated with other genes (Supplementary Fig. 1e). As a reference, we also show the comparable reduction of remaining genes if they were filtered using a standard expression threshold of TPM > 1 (13,567 ± 874 genes, Supplementary Fig. 1b).

The resulting networks consist of edges that are shared across multiple tissues (core transcriptional modules), as well as edges that are only present in a small number of tissues (tissue-specific modules). We considered edges present in less than five tissues as tissue-specific, and edges present in at least five tissues as core transcriptional modules (Supplementary Fig. 1c, d).

#### Ontology-derived functional and phenotypic similarity network

To capture gene relationships on functional and phenotypic levels, we incorporated expert curated data and systematic ontologies. To transform ontological annotations into gene-centric networks, we defined that two genes are functionally or phenotypically connected if they are semantically similar based on the corresponding ontology^71,72^ as follows:

We first compared several widely used measures of semantic similarity to ensure that the scores are robust for our purposes:

1. Information content (IC)-based similarity based on Resnik’s method^73^. The similarity of two terms is derived from their most informative common ancestor (MICA) in the ontology. Given ontology terms *t*_1_ and *t*_2_, their pairwise similarity is given by ${{{{{\rm{si}}}}}}{{{{{{\rm{m}}}}}}}_{{{{{{\rm{Resnik}}}}}}}({t}_{1},{t}_{2})={{{{{\rm{IC}}}}}}({{{{{\rm{MICA}}}}}})$, where ${{{{{\rm{IC}}}}}}(t)=-{{{{{\rm{log }}}}}}(p(t))$, *p*(*t*) represents the frequency of term *t* defined by $p(t)=\frac{{n}_{t}}{N},{n}_{t}$ denotes the number of descendants of term *t*, and *N* the number of descendants of the root term of interest in the ontology tree.
2. Information content (IC)-based similarity based on Lin’s method^74^. Unlike Resnik’s method, Lin’s similarity measure restricts the value to be in the range between zero and one, and is given by ${{{{{\rm{si}}}}}}{{{{{{\rm{m}}}}}}}_{{{{{{\rm{Lin}}}}}}}({t}_{1},{t}_{2})=\frac{2{{{{{\rm{IC}}}}}}({{{{{\rm{MICA}}}}}})}{{{{{{\rm{IC}}}}}}({t}_{1})+{{{{{\rm{IC}}}}}}({t}_{2})}\in [{{{{\mathrm{0,1}}}}}]$.
3. After collecting all pairwise term similarities for annotations of two genes, we next employed the Best-Match Average (BMA) strategy to combine them into a single gene similarity score. Their pairwise similarity of genes ${g}_{1}$ and ${g}_{2}$with *m* and *n* annotated terms, respectively, is given by ${{{{{\rm{si}}}}}}{{{{{{\rm{m}}}}}}}_{{{{{{\rm{BMA}}}}}}}({g}_{1},{g}_{2})=\frac{{\sum }_{i=1}^{m}{{{{{\rm{colmax}}}}}}({{{{{\rm{S}}}}}})+{{{{{\rm{rowmax}}}}}}(S)}{m+n},$where $S\in {{\mathbb{R}}}^{{mxn}}$ is the matrix containing the pairwise similarity values of the ontology terms associated with the two genes, ${{{{{\rm{rowmax}}}}}}(S)$ and ${{{{{\rm{colmax}}}}}}(S)$ are vectors of length *m* and *n*, containing the maximum similarity values across all rows and columns of matrix *S*.
4. Frequency-based similarity, where the similarity between two genes is given by the number of shared annotations, i.e., ${{{{{\rm{si}}}}}}{{{{{{\rm{m}}}}}}}_{{{{{{\rm{freq}}}}}}}({g}_{1},{g}_{2})=|{T}_{g1}\cap {T}_{g2}|$, where ${T}_{{gk}}$ is the set of ontology terms (including ancestor terms) associated with gene *k*.

We found that the respective similarity values are strongly correlated, indicating that the resulting networks are robust against details of the used methods (Supplementary Fig. 2a). We chose to proceed with the IC-based Resnik’s method with the Best-Match Average (BMA) combination strategy, as it has been demonstrated to both be among the simplest methods, while also providing the most reliable performances across different tasks^71,75^. We used the R packages GoSemSim^76^ and OntologyX^77^ to navigate and compute the similarity measurements.

Gene pairs with minimal similarity value, i.e., pairs whose only common annotation is the root term of the considered ontology branch (i.e., “Molecular Function” or “Biological Process”) were considered as unrelated and therefore removed from further consideration. For example, there are over 21M gene pairs connected at this level in the GO (BP) branch (similar score = 0.447, Supplementary Fig. 2b). This led to the removal of 230 genes with no commonly associated MICA with other genes beyond the root term.

All ontology-based networks (GO:BP, GO:MF, MPO and HPO) were constructed according to the following procedure summarized in Supplementary Fig. 2c: Pairwise similarity scores given by the procedures above resulted in dense weighted networks. We further applied the disparity filter^19^ to extract the backbone of the network and discard structural redundant edges (gene pairs with corresponding disparity p-value > 0.05). The disparity filter provides a dynamic cutoff that considers the strength of the similarity scores of a gene in reference with all similarity values of its neighbors. Similar to using a hard cutoff, edges between gene pairs with low similarity scores (e.g., $0 < {{{{{\rm{sim}}}}}}({g}_{1}{g}_{2}) < 3$ in GO:BP) are removed while those with high similarity scores (${{{{{\rm{sim}}}}}}({g}_{1}{g}_{2}) > 6$) are virtually unaffected. Edges with medium similarity scores ($3 < {{{{{\rm{sim}}}}}}({g}_{1}{g}_{2}) < 6$) may either remain or be discarded based on their similarity score with respect to all other connected genes (Supplementary Fig. 2d).

Overall, networks derived from semantic similarity measures favor gene pairs that are similarly annotated over highly, but diversely annotated gene pairs Supplementary Fig. 2e). Gene pairs with high similarity scores often belong to the same protein families such as the ER membrane protein complexes (EMC), olfactory receptors (OR), and membrane transporters, and tend to share a large fraction of annotated GO terms (Supplementary Fig. 2e, right). We further demonstrated this for the example of GO terms associated with TP53 (gene with highest number of publications) and TGFB1 (gene with highest number of associated GO terms). While both genes are well characterized, with 87 and 176 annotated GO terms, respectively, only ten annotations are shared, indicating that they are involved in distinct biological processes (Supplementary Fig. 2f). As a result, the computed similarity score and subsequent disparity *p*-value failed to reach the significance threshold, meaning that the two genes are not connected (Supplementary Fig. 2e). This effect is observed across most well characterized genes, leading to the slightly negative literature bias of ontology-derived networks (Fig. 1h and Supplementary Fig. 4c–f). We found that densely connected clusters within the constructed networks recapitulate biological processes corresponding to shared terms on their respective ontologies (Supplementary Fig. 3a, clusters with Bonferroni-Holm corrected enrichment hypergeometric *p*-value < 1e-20 were labeled).

#### Pathway co-membership networks

Gene-pathway associations were downloaded from the Reactome website https://reactome.org/download-data/ (accessed 25 January 2019) under the Reactome Pathways Gene Set section. For every gene pair, we collected the number of shared pathway annotations. In the pathway co-membership network construction, two genes were connected if they share at least five Reactome pathway annotations (to prevent associations due to common pathways).

#### Disparity filter

To extract the backbone of dense, weighted networks resulting from semantic and correlation-based construction, we applied a disparity filter^19^. For a given network, we computed a *p*-value for all edges between nodes *i* and *j* as ${p}_{{ij}}={(1-{w}_{{ij}})}^{k-1}$, where *w*_*ij*_ is the edge weight for node *i* normalized over all its edges, and *k* denotes its degree. We only kept edges for which both *p*_*ij*_ and ${p}_{{ji}}$ reached a threshold significance level.

All network data and corresponding details are available in Supplementary Data 1, 2.

### Measurements of network characteristics

The network characteristics shown in Fig. 1 h (number of nodes and edges, clustering and assortativity) were computed using the R package igraph^78^ (https://igraph.org).

For a global assessment of the literature bias present in a particular network we used the Spearman’s correlation coefficient between the network degree of a gene and the number of publications mentioning the gene. The number of publications was queried using the INDRA python module 78 (http://www.indra.bio, accessed on 12 April 2019), the resulting data is provided in Supplementary Data 8.

For a more local assessment of correlation structures within the connection patterns of a network, we used the local assortativity (*ρ*), a node-level property whose sum over all nodes is equal to the assortativity of the network^79^. It is defined as $\rho =\frac{j(j+1)(\bar{k}-{\mu }_{q})}{2M{{\sigma }_{q}}^{2}}$, where *j* is the excess degree, $\bar{k}$ the average excess degree, and *M* the number of edges in the network. The excess degree follows the distribution $q(k)=\frac{(k+1)p(k+1)}{\bar{k}}$. We employed the concept to demonstrate that the overall disassortativity can also be present among interactions derived from high-throughput studies such as the BioPlex network (Supplementary Fig. 3f).

### Network similarity computation and randomization

Given a pair of networks *A* and *B* with the set of edges *E*_*A*_ and *E*_*B*_ respectively, we quantified the network similarity using the edge overlap index (*S*_*AB*_):

$${S}_{{AB}}=\frac{{{{{{{\rm{|}}}}}}E}_{A}\cap {E}_{B}{{{{{\rm{|}}}}}}}{{{{{{\rm{min }}}}}}({{{{{{\rm{|}}}}}}E}_{A}{{{{{\rm{|}}}}}},{{{{{\rm{|}}}}}}{E}_{B}{{{{{\rm{|}}}}}})}$$

We used a dissimilarity measure defined as ${d}_{{AB}}=1-{S}_{{AB}}$ to construct a 2D map that preserves network dissimilarities by employing Kruskal’s non-metric multidimensional scaling (R package MASS). Finally, we compared the measured similarity of each network pair to random expectation: For each network, we performed 10 permutations of node indices, resulting in 100 permutations for a network pair, which we used as random reference distribution to assess the measured overlap similarity. We then computed the *z*-score and corresponding empirical *p*-value. A network pair with *p-*value < 0.05 is considered significantly similar (Supplementary Fig. 3c, d).

### Characterization of co-expression network with essentiality data

We characterized our tissue-specific co-expression networks constructed based on GTEx expression data as follows: We hypothesized that genes that are highly co-expressed across several tissues are likely required for cellular development and survival, and should show a strong tendency of being essential genes. To test this hypothesis, we used the list of human essential genes from the OGEE database (v2, retrieved on 16 April 2019. Supplementary Data 9).

### Rare genetic disease gene association data

The structure of the Orphanet Rare Disease Ontology was queried and processed using the R interface of the Ontology Lookup Service (https://lgatto.github.io/rols/index.html). We considered all descendant terms of “Rare genetic disease” (Orphanet:98053) that were associated with at least 20 genes, resulting in 26 rare genetic disease groups. The disease groups and all disease-gene associations can be found in Supplementary Data 5).

### Disease-network landscapes via node2vec embedding algorithm

To visualize large (genome-scale) networks where the modularity can be difficult to observe, we employed the python3 implementation of the node2vec graph embedding algorithm^29^ (https://github.com/eliorc/node2vec). Nodes were embedded into 64-dimensional Euclidean space and subsequently projected on a 2D plane using t-SNE^80^ (Supplementary Fig. 6c). Note that the predictions in this work were performed on the original network space as the resulting coordinates in the embedded Euclidean space are subject to the parameterization in both the node embedding and the dimensionality reduction. Since different node embedding techniques and parameter sets may preserve different topological structures^81–83^, their reliability may vary depending on the particular machine learning task^84^.

### Identification of the significance of a disease module

The size of the largest connected component of random subsets of *m* nodes in a network is expected to follow a normal distribution, provided that *m* is larger than the percolation threshold. We can therefore empirically estimate the significance of a given module size by the *z*-score and corresponding *p*-value compared to randomly selected nodes. Networks in which the size of the largest connected component of the genes associated with a particular disease exceeded a threshold of *p*-value < 0.05 (after Benjamini–Hochberg correction) were considered significant.

### Gene ID mapping, homolog conversion, and enrichment analysis

All human gene identifiers from different resources were mapped to NCBI standard symbols. For mouse to human gene mapping, we used the Moue Genome Informatics homologs mapping http://www.informatics.jax.org/downloads/reports/index.html.

Gene enrichment results were queried using EnrichR^85^.

### Informed multiplex network propagation algorithm

The standard multiplex network propagation is defined by an equal probability for the random walker to visit any neighbor from the current layer *m* or any other layer *n*^86^. For *L* network layers with *N* nodes each, this can be represented through the supra-adjacency matrix $S\in {{\mathbb{R}}}^{{NL}\times {NL}}$:

$${{{{{\rm{S}}}}}}=\left[\begin{array}{cccc}{A}_{1} & {{{{{\rm{I}}}}}} & \ldots & {{{{{\rm{I}}}}}}\\ {{{{{\rm{I}}}}}} & {A}_{2} & \ldots & {{{{{\rm{I}}}}}}\\ \vdots & \vdots & \ddots & \vdots \\ {{{{{\rm{I}}}}}} & {{{{{\rm{I}}}}}} & \ldots & {A}_{L}\end{array}\right]$$

where *A*_*m*_ is the adjacency matrix for network layer *m* ($m\in \{1...L\}$) and I denotes the identity matrix.

We extended this standard algorithm towards an informed propagation method where the walker visits more relevant layers with higher probability. We quantify the relevance of a network *m* for a disease group *d* by the corresponding *z*-score ${z}_{{dm}}$ of the largest connected component of associated genes. We considered all network layers with ${z}_{{dm}}\ge 1.645$ (corresponding to the 95% confidence level under normal distribution) as informative and defined the relevance score (${\pi }_{{dm}}$) as the normalized *z*-score across all informative layers:

$${\pi }_{{dm}}={z}_{{dm}}/\mathop{\sum}\limits_{m}{z}_{{dm}}$$and $$\mathop{\sum}\limits_{m}{\pi }_{{dm}}=1$$

The relevance score ${\pi }_{{dm}}$ was then used to determine the transition probability $p({m|n})$ between layers *n* an *m*, so that the walker visits more informative layers with a higher probability corresponding to their respective ${\pi }_{{dm}}$ values. This is achieved by employing the concept of reversible Markov chain Monte Carlo that requires the following detailed balance condition:

$${\pi }_{m}p(m{{{{{\rm{|}}}}}}n)={\pi }_{n}p(n{{{{{\rm{|}}}}}}m)$$

To satisfy this condition, we define $p({m|n})=\frac{1}{L}{\min }(1,\frac{{\pi }_{m}}{{\pi }_{n}})$ and $p({m|m})=1-\mathop{\sum}\limits_{n\ne m}p({m|n})$. The informed supra-adjacency matrix $\widetilde{{{{{{\rm{S}}}}}}}$ can thus be written as

$$\widetilde{{{{{{\rm{S}}}}}}}={{{{{\rm{p}}}}}}\circ {{{{{\rm{S}}}}}}=\left[\begin{array}{cccc}{p}_{11}{{{{{{\rm{A}}}}}}}_{1} & {p}_{12}{{{{{\rm{I}}}}}} & \ldots & {p}_{1L}{{{{{\rm{I}}}}}}\\ {p}_{21}{{{{{\rm{I}}}}}} & {p}_{22}{{{{{{\rm{A}}}}}}}_{2} & \ldots & {p}_{2L}{{{{{\rm{I}}}}}}\\ \vdots & \vdots & \ddots & \vdots \\ {p}_{L1}{{{{{\rm{I}}}}}} & {p}_{L2}{{{{{\rm{I}}}}}} & \ldots & {p}_{{LL}}{{{{{{\rm{A}}}}}}}_{{{{{{\rm{L}}}}}}}\end{array}\right]$$

Finally, we incorporate the informed supra-adjacency matrix into the random walk with restart algorithm:

$${p}_{t+1}=(1-r)\widetilde{{{{{{\rm{S}}}}}}}{p}_{t}+r{p}_{0}$$

where ${p}_{0}$ is the initial visiting probability vector with ${p}_{0}(i)=1/k$ if node *i* is one of *k* seed nodes, and ${p}_{0}(i)=0$ otherwise. ${p}_{t}$is the visiting probability at iteration step *t*, and $r\in [{{{{\mathrm{0,1}}}}}]$ is the restart probability. In this analysis, we chose *r* = 0.7.

The final visiting probability (${p}_{{{{{{\boldsymbol{\infty }}}}}}}$) can be obtained numerically when the convergence criteria are met ($|{p}_{t+1}-{p}_{t}|=0$). The visiting probability of a node is the arithmetic mean of the visiting probability across all layers. In retrieval tasks, nodes are ranked based on this final visiting probability. Seed nodes are omitted from the ranking.

### Cross-validation performance assessment

The prediction performance was assessed using 10-fold cross-validation for retrieving genes associated with individual rare disease groups. Area under the receiver operating characteristic curve (AUROC) computations and plots were performed using the cvAUC and pROC packages in R. Differences between ROCs were evaluated using the two-sided DeLong’s test^87^.

We first considered four different settings: (1) baseline single layer (the PPI), (2) the most relevant single layer for each disease according to the lowest LCC z-score, (3) all network layers, and (4) all relevant network layers, i.e., those with a significant LCC *z*-score for the disease (*p*-value < 0.05, Benjamini–Hochberg correction for multiple hypotheses).

To further investigate the contribution of individual layers, as well as potential curation biases on the overall predictive power, we performed several additional benchmarks on different subsets of the multiplex network:

We first analyzed the impact of interactions curated from small-scale experiments on the prediction performance of the PPI network layer (Supplementary Fig. 4a). To this end, we considered two subsets of the full PPI, an unbiased subset consisting of interactions from systematic high-throughput studies, and a curated subset consisting of all other interactions (see above). The unbiased PPI contributes to 13% of all interactions in the full PPI, and, as expected, shows a less pronounced literature bias (Supplementary Fig. 6b). While the curated PPI performs equally well as the full PPI in the disease gene prediction task, the performance of the unbiased PPI drops significantly (median AUROC = 0.62, *p*-value = 1.76e-9, FDR-corrected Durbin-Conover non-parametric test, Supplementary Fig. 7c). To assess the extent to which the reduced size of the unbiased PPI contributes to this drop, we repeated the analysis on ten random subsets of the curated PPI that are of the same size as the unbiased PPI subset. We found that these random subnetworks have a performance comparable to the one of the unbiased PPI (with a median AUROC of 0.58 even slightly reduced, Supplementary Fig. 7c). This indicates that the performance of the PPI network is mainly driven by its size, rather than details of the interaction curation. This, in turn, suggests that confirmatory biases that may result from including curated interaction data are likely to play only a minor role for the overall performance, at least for PPI data.

We next assessed the prediction performance of the multiplex network upon removing other network layers derived from curated databases, specifically the layers based on shared pathway membership, phenotypic similarity (HPO and MPO), and GO (BP and MF) similarity. We first computed the 10-fold cross-validation AUROC after removing each of these layers individually. For most layers, we observed only a slight drop in the performance (median AUROC between 0.87 and 0.88; Supplementary Fig. 7d), indicating that the core connectivity of disease genes across different layers is robust against the removal of individual layers. The only layer with a stronger impact is the HPO phenotype layers, whose removal resulted in a reduction of AUROC to 0.80 (*p*-value = 0.0003, FDR-corrected Durbin-Conover non-parametric test). This is not unexpected given the strong predictive power of phenotypes as close proxies to diseases which forms the basis for their usage in clinical settings and is documented in the literature^1,2^, as well as in the gene-level benchmarks discussed in the patient candidate gene prioritization below.

Finally, we determined the predictive performance of the multiplex network after removing all layers that involve curated data (Reactome, GO, HP, MP, and PPI), leaving only relevant co-expression and co-essentiality networks for the propagation. While these high-throughput data alone do carry predictive power, their performance was significantly lower compared to using all available data sources (AUROC = 0.71, *p*-value = 1.17e-11). Interestingly, we also observed an occasional increase in performance, such as for rare genetic endocrine diseases, one of the worst performing disease groups in the reference setting (AUROC increased from 0.64 to 0.71). The propagation only took place on the adipose tissue co-expression network (ADS), which, in addition to its traditional role for excess lipid storage, has recently been recognized as an endocrine organ^16,17^.

Taken together, these results suggest that the predictive power of the multiplex network can be best understood as a collective characteristic of all disease relevant layers, rather than being primarily driven by specific individual layers.

### Cohort of patients with intellectual disability

We first developed and tested our method on a locally available, well-controlled cohort of patients with intellectual disability (ID), before applying it to a much larger cohort obtained from the RD-Connect Genome-Phenome Analysis Platform (GPAP)^88^. To conduct a temporal-holdout benchmarking, we also curated a subset of the RD-Connect cohort containing patients with causal genes discovered after all data used in the network construction was retrieved. The details of the three cohorts are as follows:

#### Local cohort

We gained access to variant data from eight patients with confirmed causal gene (two females and six males aged between three to twenty years old; see Supplementary Data 11 for details). The recruitment was based on the referral by clinicians, with the purpose of genetic testing and there was no compensation involved. Informed consents were signed by the patients or their legal guardians and the processes were reviewed by Ethics Committee of the Medical University of Vienna; and/or Haunerschen Kinderspital, Munich, Germany; Servicio di Consulenza Genetica, Bolzano, Italy; University Hospital Zagreb, Zagreb, Croatia; General Hospital Varazdin, Varazdin, Croatia; and Tehran University of Medical Sciences, Tehran, Iran in accordance with the Declaration of Helsinki. All patient variant data were obtained from exome-sequencing performed at the Biomedical Sequencing Facility (BSF) at the CeMM Center for Molecular Medicine of the Austrian Academy of Sciences (CeMM). Genomic DNA was extracted (QIAamp DNA Mini Kit, Qiagen) from whole blood from patients, parents and participating siblings. Quantity and quality of patient DNA were validated by Qubit 2.0 Fluorometric Quantitation system (Life Technologies). Exome libraries were prepared using the Nextera DNA Flex Exome Library Prep Kit (Illumina). Genomic DNA was tagmented, size-selected and amplified followed by two rounds of hybridization with biotinylated baits and capture with streptavidin-conjugated magnetic beads. After enrichment, library fragments representing in total 45 Mb coding region were amplified and size-selected. Final library pools were quality controlled and sequenced on a HiSeq 3000 instrument (Illumina) using 75 bp paired-end chemistry. DNA sequences were mapped to GRCh37 (hg19) version of human reference genome using Burrows-Wheeler Aligner with default parameters. Single nucleotide variants (SNVs) and indels were annotated with gnomAD^89^, CADD-Phred^90^, dbSNP^91^ and ClinVar^92^ data. Subsequent filtering of remaining variants of interest was based on the inheritance pattern, variant type (high or moderate impact as classified by Ensembl database), allele frequency (<1%) in gnomAD database, and gene lists of interest in relation to the patient’s symptoms annotated by Human Phenotype Ontology (HPO).

Genes associated with HPO terms describing a patient’s major symptoms were used as patient-specific seed genes (Supplementary Fig. 7 f), weighted by the frequency of association, i.e., a gene will be given a higher weight if it is associated with more than one phenotype found in the patient. After standard methods of filtering for high confidence variants were exhausted, up to 46 candidate genes remained, with an average number of 16 candidate genes per patient. Our patient-specific multiplex network propagation ranked the validated causal gene first in four cases, in all cases it was among the top five predictions (Supplementary Fig. 7g). Strikingly, the algorithm correctly pinpointed the causal gene in the two most complex cases, where patients presented with high confidence variants from 46 and 33 genes, respectively.

#### RD-Connect cohort

To overcome the small number of patients available in our local cohort, we have gained access to RD-Connect Genome-Phenome Analysis Platform (GPAP), one of the largest global infrastructures for storing and sharing genotype and phenotype data of rare disease patients (https://platform.rd-connect.eu/)^88^. To match our local cohort, we queried patients whose phenotypes are characterized by intellectual disability (HPO term HP:0001249). Of the resulting 819 patients, 131 were solved cases, i.e., patients with a confirmed causal variant that could thus be utilized for benchmarking (Fig. 6a). The inclusion of these patients expanded the original sample size by a factor of over 16. The variants were filtered for highly stringent pathogenicity include these following tools and criteria: (1) Variant type: SNV, (2) SNV effect prediction: Mutation Taster—A (Annotated and disease causing) and D (Disease causing); PolyPhen2—D (Possibly damaging) and P (Possibly damaging); SIFT—D (Damaging), CADD score ≥ 20, (3) Minor Allele Frequency: gnomAD allele frequency <  0.01; 1000Genome Protect AF < 0.01.

#### Temporal-holdout benchmarking cohort

All curated databases (GO, MPO, HPO, and the PPI) were retrieved before March 2019, we thus sought to filter for patients with causal genes that were discovered only after that point in time (Supplementary Fig. 8a). To this end, we collected the list of confirmed intellectual disability (ID) causal genes from Genomics England PanelApp^93^, a large expert reviewed platform for disease gene causality evaluation (https://panelapp.genomicsengland.co.uk/panels/285). We downloaded the ID panel v3.0, which has the signed off date of 10/12/2019 and consists of 1,085 confirmed ID genes. Within our cohort of 131 RDconnect patients, 21 had causal genes not included in this panel gene list. These genes can thus be considered to have been unknown to the expert community at the time of network curation. By restricting our validation analysis to these 21 causal genes, we can assume that their disease association is not implicitly contained in the data that we use in the prediction.

### Gene-level ranking benchmark

As a benchmark for the network-based informed multiplex propagation for patient candidate gene prioritization, we also implemented several ranking methods relying solely on gene-based features. Specifically, we employed the same gene features that were used to construct the multiplex networks: (1) pathway information—ranking genes involved in more pathways higher; (2) expression level information—ranking genes with higher expression levels in brain tissues higher; (3) general literature counts—ranking genes linked to a higher number of publications higher; (4) phenotypic similarity—ranking genes higher that are associated with Human Phenotype Ontology (HPO) terms described in a patient.

### Reporting summary

Further information on research design is available in the Nature Research Reporting Summary linked to this article.

## Supplementary information

Supplementary Information

Reporting Summary

Description of Additional Supplementary Files

Supplementary Data 1

Supplementary Data 2

Supplementary Data 3

Supplementary Data 4

Supplementary Data 5

Supplementary Data 6

Supplementary Data 7

Supplementary Data 8

Supplementary Data 9

Supplementary Data 10

Supplementary Data 11

## Data Availability

Data generated in this study are provided in the Supplementary Information/Source Data file. The RDconnect Genome-Phenome Analysis Platform (GPAP) data are available under restricted access, which can be obtained by validated users via the platform at https://platform.rd-connect.eu/.

## Code availability

Source code and cache data is available at the https://github.com/menchelab/MultiOme^94^. The supplementary Explorer app for detailed inspection of disease-network specificity is available at www.menchelab.com/MultiOmeExplorer.
