# Supplementary material for: Network analysis reveals rare disease signatures across multiple levels of biological organization
Source: Nat Commun. 2021 Nov 9;12:6306. doi: 10.1038/s41467-021-26674-1 (PMC8578255; doi:10.1038/s41467-021-26674-1)
Supplement: Supplementary file 1 — Supplementary Information [file 41467_2021_26674_MOESM1_ESM.pdf]

Supplementary Figure 1

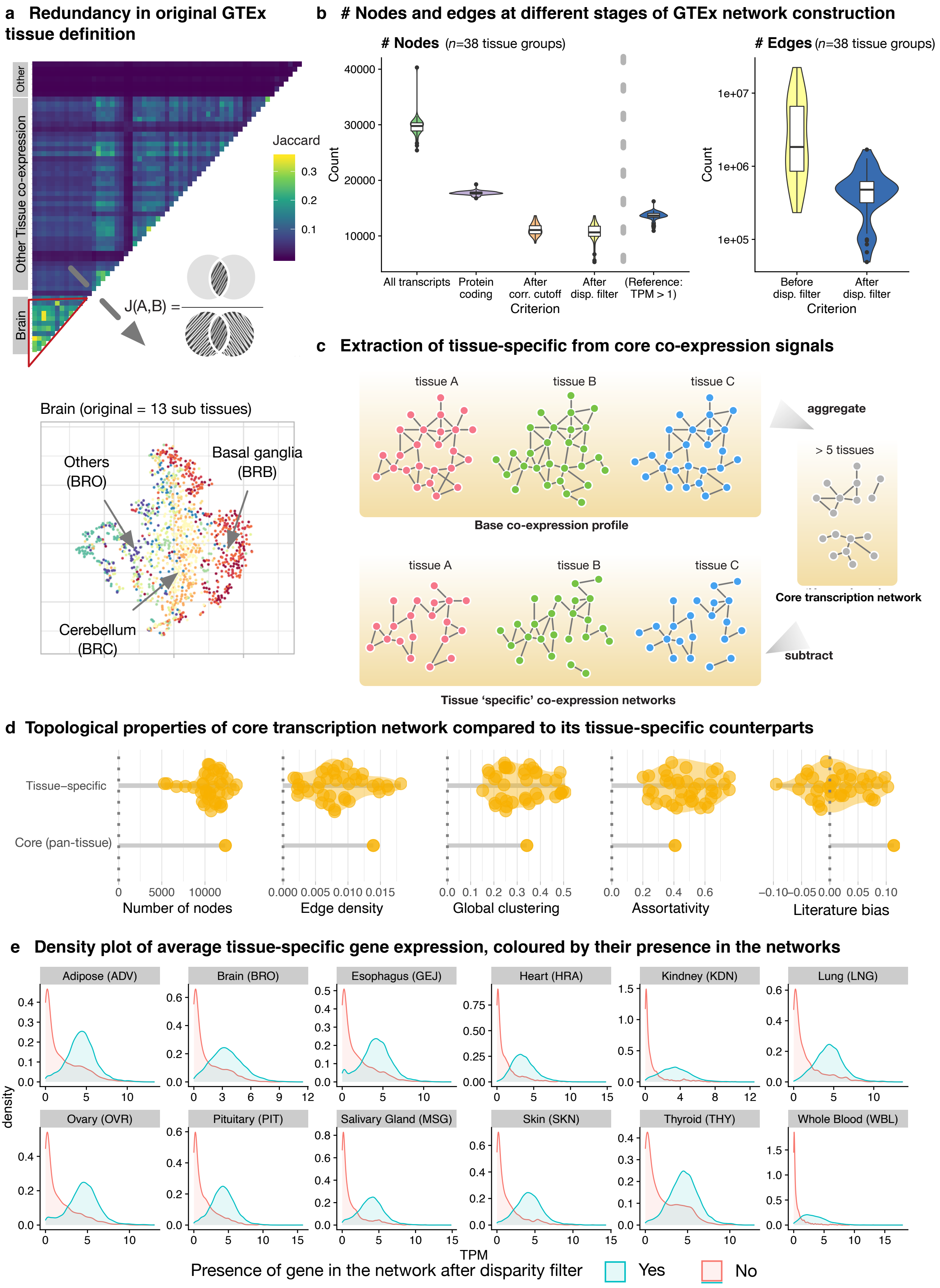

Supplementary Figure 1. Co-expression network construction. (a) Heatmap with pairwise Jaccard similarity between co-expression matrices of all GTEx tissues. Inset shows the t-SNE projection of expression profiles in 13 brain tissues, which were merged into three groups based on anatomical and expression similarity to reduce redundancy. In total, the process resulted in 38 tissue groups. **(b)** Left: number of genes for each step of the co-expression network construction process. Right: number of edges before and after applying the disparity filter (n=38 tissue groups). The network-based method removed a large number of spurious correlations (median number of interactions before and after = 1.83e6 and 4.78e5, respectively), while only slightly decreasing the number of genes. Bounds of box represent 25th and 75th percentiles, center the median, whiskers 10th and 90th percentiles, respectively. **(c)** Extracting tissue-specific co-expression networks by separating all edges observed in five or more tissues into an own core transcriptional network layer. **(d)** Topological properties of the core transcription layer, consisting of 12,364 nodes and 1,062,924 edges, compared to its tissue-specific counterparts. **(e)** Distribution of gene expression levels in the tissue-specific networks. The dynamic cutoff of the disparity filter generally tends to remove lowly expressed genes and keep highly expressed genes, while also allowing for the inclusion of genes that are lowly expressed, yet strongly correlated with other genes.

Supplementary Figure 2

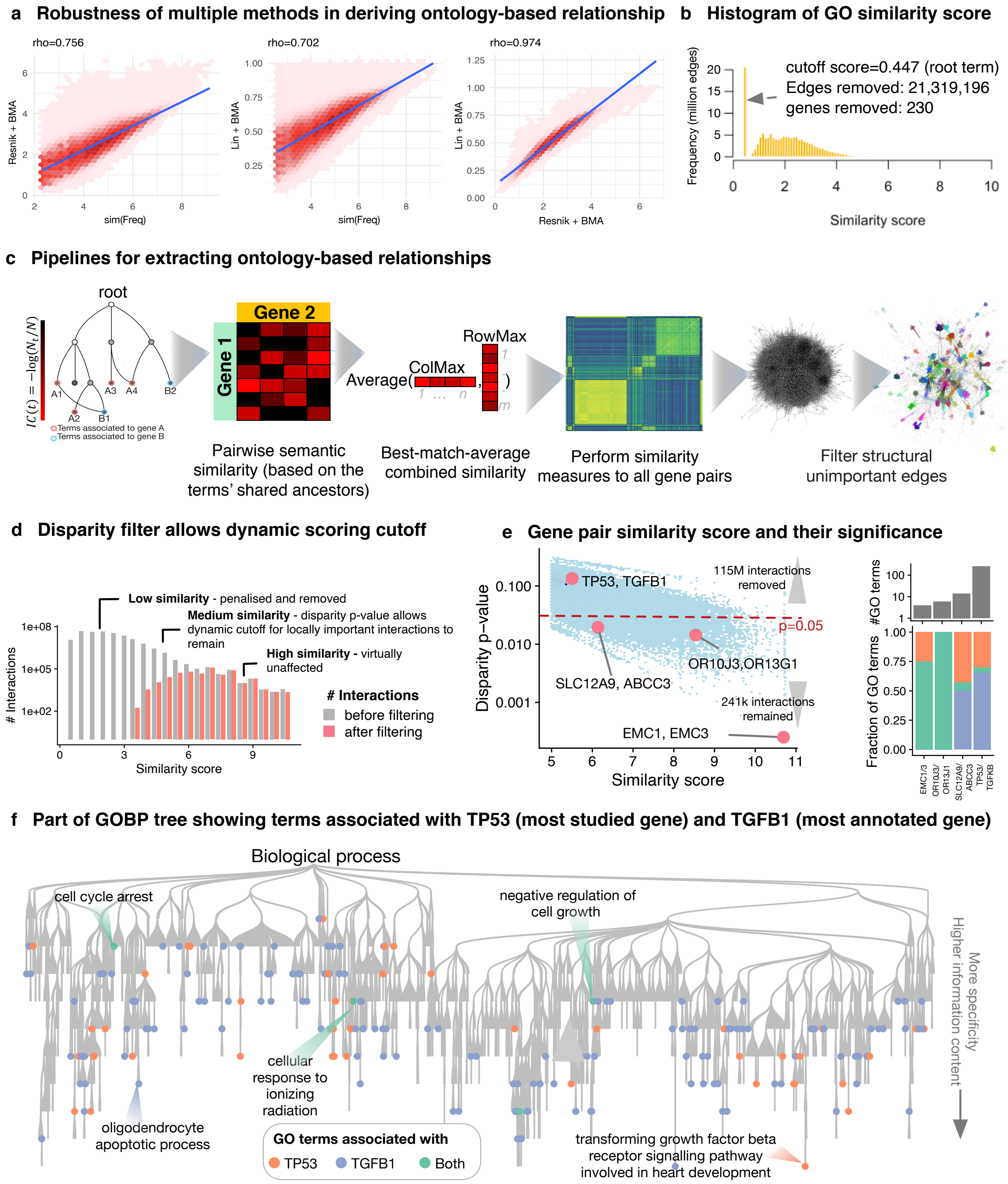

Supplementary Figure 2. Construction of the ontology-derived networks. (a) Pairwise comparison between different pairwise similarity measures (IC-based and frequency-based). The strong correlations show the robustness of ontology-based gene pair relationships. **(b)** Distribution of pairwise similarity of GO biological process annotations over all gene pairs. We filtered out all gene pairs with minimal similarity, i.e. those for which the only common ancestor is the root term of the ontology tree. **(c)** Overall pipeline for extracting ontology-based relationships. We measured IC-based semantic similarity of all gene pairs, then removed weak and redundant edges using the disparity filter. **(d)** Distribution of pairwise similarities of GO biological process annotations across all gene pairs before and after applying the disparity filter. The disparity filter corresponds to a dynamic cutoff. While gene pairs with low similarity scores are generally removed and pairs with high similarity scores remain virtually unaffected, gene pairs with medium similarity scores either remain or are discarded depending on the strength of the similarity scores with all involved neighbors. **(e)** Relationship between disparity filter p-value (see Methods for details) and similarity score according to GO biological process annotations for all gene pairs. Networks derived from semantic similarity measures favor gene pairs with similar annotation depths (e.g., from the same protein families) over highly, but diversely annotated gene pairs. Gene pairs with high similarity scores often belong to the same protein family such as the ER membrane protein complexes, olfactory receptors, and membrane transporters, and tend to share a large fraction of annotated GO terms. The barplot on the right shows the GO terms shared by four example gene pairs (green indicates shared terms, blue and red terms unique to the first and second gene, respectively). **(f)** Portion of the biological process branch of the GO highlighting terms annotated to TP53 (most studied gene) and TGFB1 (most annotated gene). While both genes are well characterized, with 87 and 176 annotated GO terms, respectively, only ten annotations are shared, indicating that they are involved in distinct biological processes. As a result, the computed similarity score and subsequent disparity *p*-value failed to reach the significance threshold, meaning that the two genes are not connected.

Supplementary Figure 3

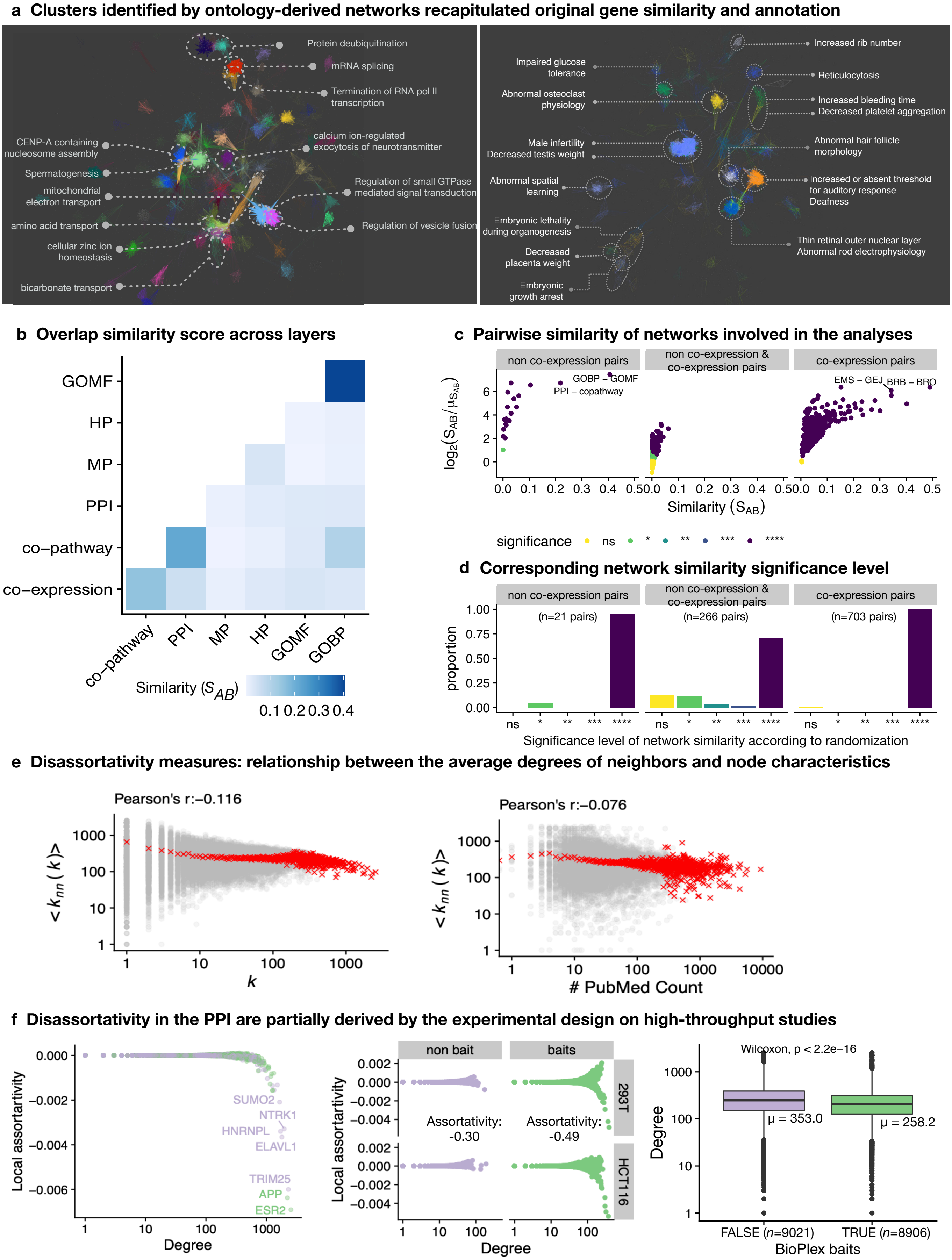

Supplementary Figure 3. Network properties and characterization. (a) Visualizations of the GO biological process (left) and mammalian phenotypic similarity (right) network layers with clusters highlighted that correspond to significantly enriched network communities (clusters with Benjamini-Hochberg corrected enrichment hypergeometric p-value < 1e-20 were labelled). **(b)** The pairwise edge overlap (S_{AB}) between the different layers is generally low, indicating that each layer provides different pieces of information and that the overall redundancy between the layers is low. **(c)** Pairwise similarity between all networks. The x-axis shows similarity scores (S_{AB}) of network pairs, the y-axis represents the effect size as measured by log fold-change of S_{AB} relative to the expected value from randomized network pairs of the same sizes. The panels show the values of non co-expression layers (n=21), non-coexpression and co-expression (n=266), and among co-expression network pairs (n=703), from left to right, respectively. **(d)** Bar charts summarizing the empirical significance of pairwise network similarity in (c), showing that the overlap is larger than expected by chance, in particular among the co-expression layers. This indicates that certain biological mechanisms are represented across different network layers. (Randomization p-values threshold: p<0.05:*, p<0.01:**, p<0.001:***, p<0.0001:****, one-sided Z-test, see Methods). **(e)** Average degree of neighbors of a gene vs. its degree (left) and PubMed count (right). Grey dots show the values for each gene, red dots the averages. Peripheral nodes (low k) tend to connect to nodes of higher degree, and vice versa, resulting in a weak overall correlation (Pearson's r = -0.12). Similarly, more studied genes tend to be connected to lower degree genes (Pearson's r = -0.08). **(f)** Local assortativity of genes within the BioPlex network for bait proteins (green) and prey proteins (purple). Left: The relationship between local assortativity score and network degree of the BioPlex network reveals disassortativity among hubs. Middle: Similar analysis, measuring interactions from the two cell lines used in the experiments separately, further revealed that bait proteins contribute to the overall disassortativity of the network. Right: Comparison of the average degree of neighbors between the bait and prey proteins: Bait proteins show lower average degree of neighbors than prey proteins (with degrees of 258 and 353; n=8906 and 9021 proteins, respectively; p-value < 2.2e-16, two-sided Wilcoxon test) and contribute more to the overall disassortativity of the network (degree assortativity = -0.49 and -0.30 for bait and prey proteins, respectively). Bounds of box represent 25th and 75th percentiles, center the median, whiskers 10th and 90th percentiles, respectively

Supplementary Figure 4

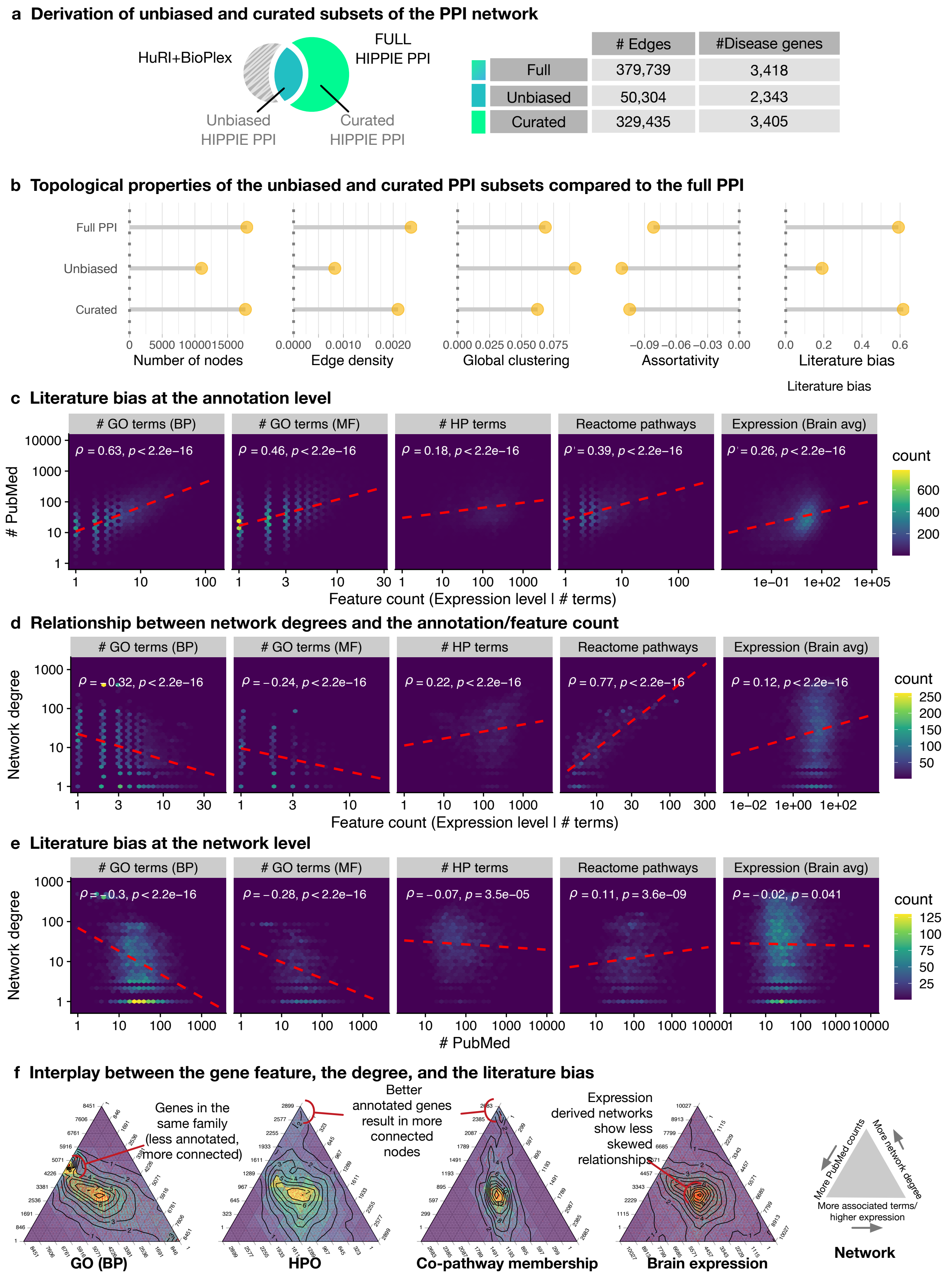

Supplementary Figure 4. Interplay between data curation, literature bias and network characteristics. **(a)** Splitting the PPI network into two categories to investigate the impact of interactions curated from small-scale experiments on the prediction performance: the unbiased PPI with interactions from systematic high-throughput screens (Y2H-based HuRI and MS-based BioPlex), and the curated PPI for the rest. The unbiased and curated categories make up 13% and 87%, respectively, of the edges contained in the full PPI. **(b)** Topological properties of the PPI subsets compared to the full PPI. **(c)** Literature bias at the annotation level. The number of publications and gene features (expression level or annotation terms) are generally positively correlated, in particular for GO annotations. Data are represented as density. Red dashed line shows line of best fit, with Spearman correlation coefficient and the corresponding p-value (Fisher z-transformation, two-sided). **(d)** Relationship between network degree and number of annotations. **(e)** Literature bias at the network level. The correlation is reversed for the GO layer due to the similarity measurement (see Supplementary Fig. 2). **(f)** Ternary plots showing the interplay between number of annotations, PubMed count and network degree. The skew of the data from the center towards an edge or a corner represents anomalies (correlation) of these features. In the case of GO, hubs tend to emerge from groups of functionally similar, and in most cases, less diversely annotated genes. For example, the region with high density of genes towards the middle of the left edge represents a group of genes with high degree and average publication counts.

Supplementary Figure 5

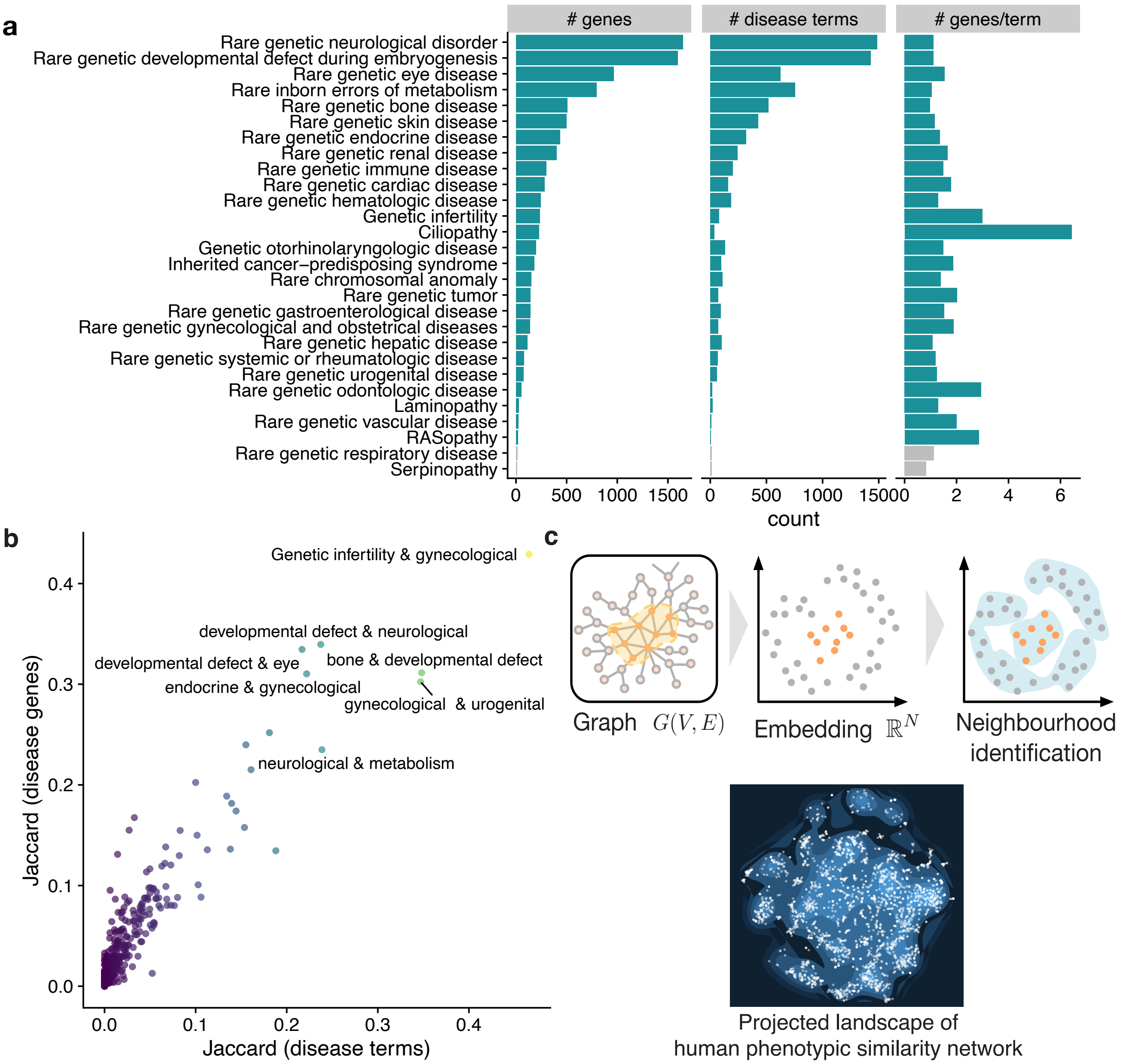

Supplementary Figure 5. Characterization of Orphanet disease groups. (a) Number of genes (left), individual disease term (middle) and their ratio (right) for the considered 26 disease groups. Grey bars represent terms with insufficient gene set size (i.e., less than 20) which were discarded from the analysis. Despite the wide range of the number of associated genes in different disease groups, the respective gene/term ratio remains consistent across all groups. **(b)** Pairwise similarity of disease groups as measured by the Jaccard index for overlapping descendant terms (x-axis) and overlapping annotated genes (y-axis). For example, rare genetic infertility is most closely related to rare genetic gynecological diseases. Overall, the disease groups are uniquely defined, with 90.5% of disease pairs having a Jaccard Index for shared genes < 0.1. **(c)** Illustration of the construction of the network landscapes using the node2vec graph embedding algorithm followed by t-SNE projection onto 2d Euclidean space. White dots are disease genes, their positions reflect their connectivity on the corresponding network. We utilize this method to visually inspect large networks where their modularity can otherwise be difficult to observe.

Supplementary Figure 6

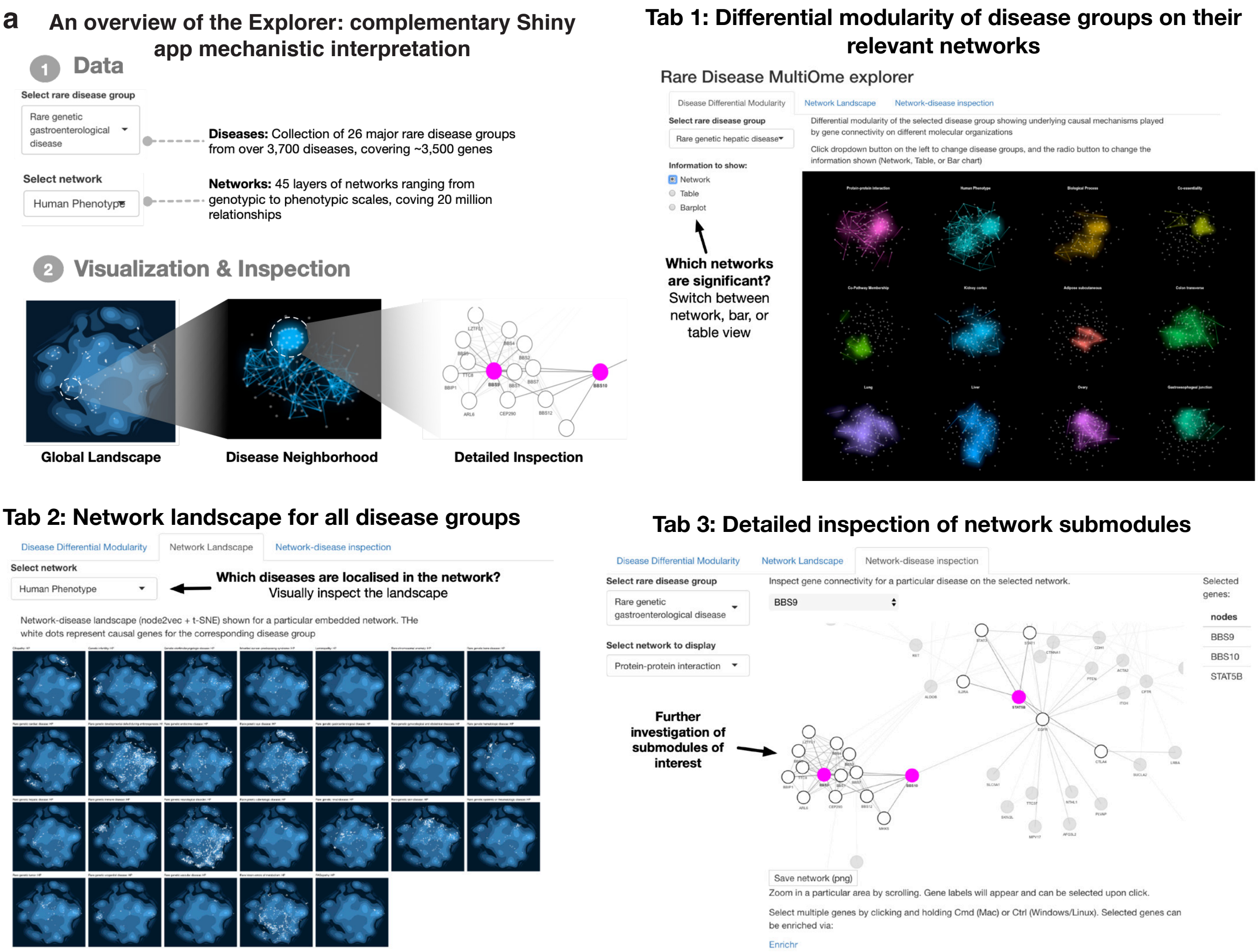

b Mechanistic interpretation for different sub-modularities for rare gastroenterological diseases

1 Identify genes representing LCC on network layers of interest

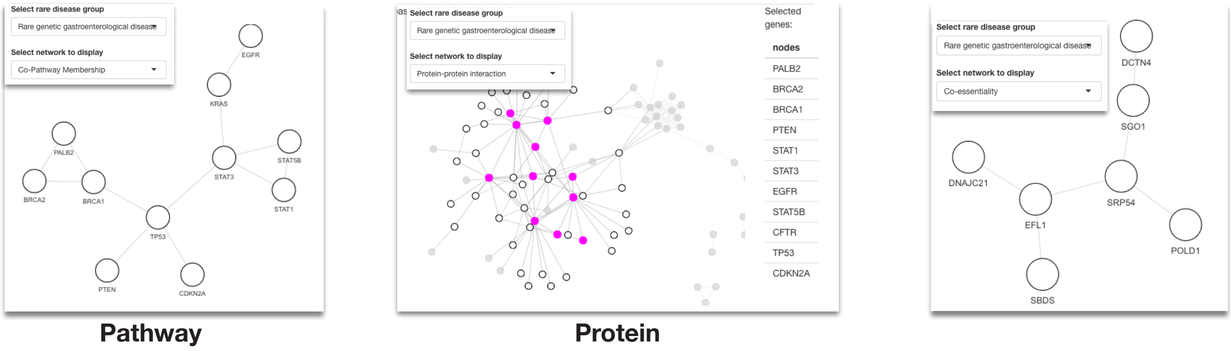

2 Performing enrichment on respective databases to gain mechanistic insights

Index	Name	P-value	Adjusted p-value
1	Downstream signal transduction Homo sapiens R-HSA-186763	1.011e-8	7.614e-7
2	Signaling by SCF-KIT Homo sapiens R-HSA-1433557	7.585e-9	7.614e-7
3	Signaling by FGFR1 in disease Homo sapiens R-HSA-5655302	3.620e-9	7.614e-7
4	Signaling by PDGF Homo sapiens R-HSA-186797	1.492e-8	8.430e-7
5	Signaling by FGFR in disease Homo sapiens R-HSA-1226099	2.901e-8	0.000001311
6	Signaling by cytosolic FGFR1 fusion mutants Homo sapiens R-HSA-1839117	1.005e-7	0.000003787
7	Diseases of signal transduction Homo sapiens R-HSA-5663202	2.574e-7	0.000007271
8	Growth hormone receptor signaling Homo sapiens R-HSA-982772	2.489e-7	0.000007271
9	FGFR1 mutant receptor activation Homo sapiens R-HSA-1839124	5.516e-7	0.00001385
10	Signaling by Interleukins Homo sapiens R-HSA-449147	0.000001182	0.00002672

Reactome

Name	P-value	Adjusted p-value
BAX complex	2.355e-14	8.429e-12
Survivin complex	4.683e-14	8.429e-12
Fatty acid synthase complex	1.074e-12	1.288e-10
BCL-2 complex	2.101e-12	1.890e-10
Bcl-2 family protein complex	7.958e-12	5.730e-10
PTEN phosphatase complex	3.000e-11	1.800e-9
B cell receptor complex	5.997e-11	3.084e-9
Phosphatidylinositol phosphate phosphatase complex	7.516e-11	3.382e-9
DNA polymerase processivity factor complex	8.080e-10	2.909e-8
PCNA complex	8.080e-10	2.909e-8

Jensen Compartments

Index	Name	P-value	Adjusted p-value
1	ribosome assembly (GO:0042255)	0.0001603	0.009775
2	nucleus localization (GO:0051647)	0.002098	0.03113
3	centromeric sister chromatid cohesion (GO:0070601)	0.002098	0.03113
4	SRP-dependent cotranslational protein targeting to membrane, translocation (GO:0006616)	0.002098	0.03113
5	nuclear migration (GO:0007097)	0.003146	0.03113
6	fatty acid homeostasis (GO:0055089)	0.003146	0.03113
7	intracellular protein transmembrane transport (GO:0065002)	0.003844	0.03113
8	establishment of protein localization to endoplasmic reticulum (GO:0072599)	0.005239	0.03113
9	mitotic sister chromatid cohesion (GO:0007064)	0.005239	0.03113
10	guanosine-containing compound metabolic process (GO:1901068)	0.005587	0.03113

Go Biological Process

Supplementary Figure 6. Overview of the MultiOmeExplorer web app (www.menchelab.com/ MultiOmeExplorer)**. (a)** The interactive Shiny app provides visualizations of all network-disease relationships that were considered in this study and allows for detailed inspection of individual disease modules of interest. The first tab gives an overview of the connection patterns of a particular disease on all network layers that were identified as significant. The second tab shows the landscape of all rare disease groups for a selected network layer. The third tab allows for a detailed inspection of submodules of interest and extract genes for downstream enrichment analysis. **(b)** Example usage for interpreting significant connection patterns among rare gastroenterological diseases. On pathway and protein levels, genes connected within the respective LCCs correspond to genes causing pancreatic carcinoma. These genes are enriched for apoptotic processes such as Bax and Survivin complexes, and major cancer pathways such as SCF-KIT, FGFR1, and PDGF. On the co-essentiality level, the LCC represents causal genes of Shwachman-Diamond syndromes, where they are co-dependent for ribosome biogenesis.

Supplementary Figure 7

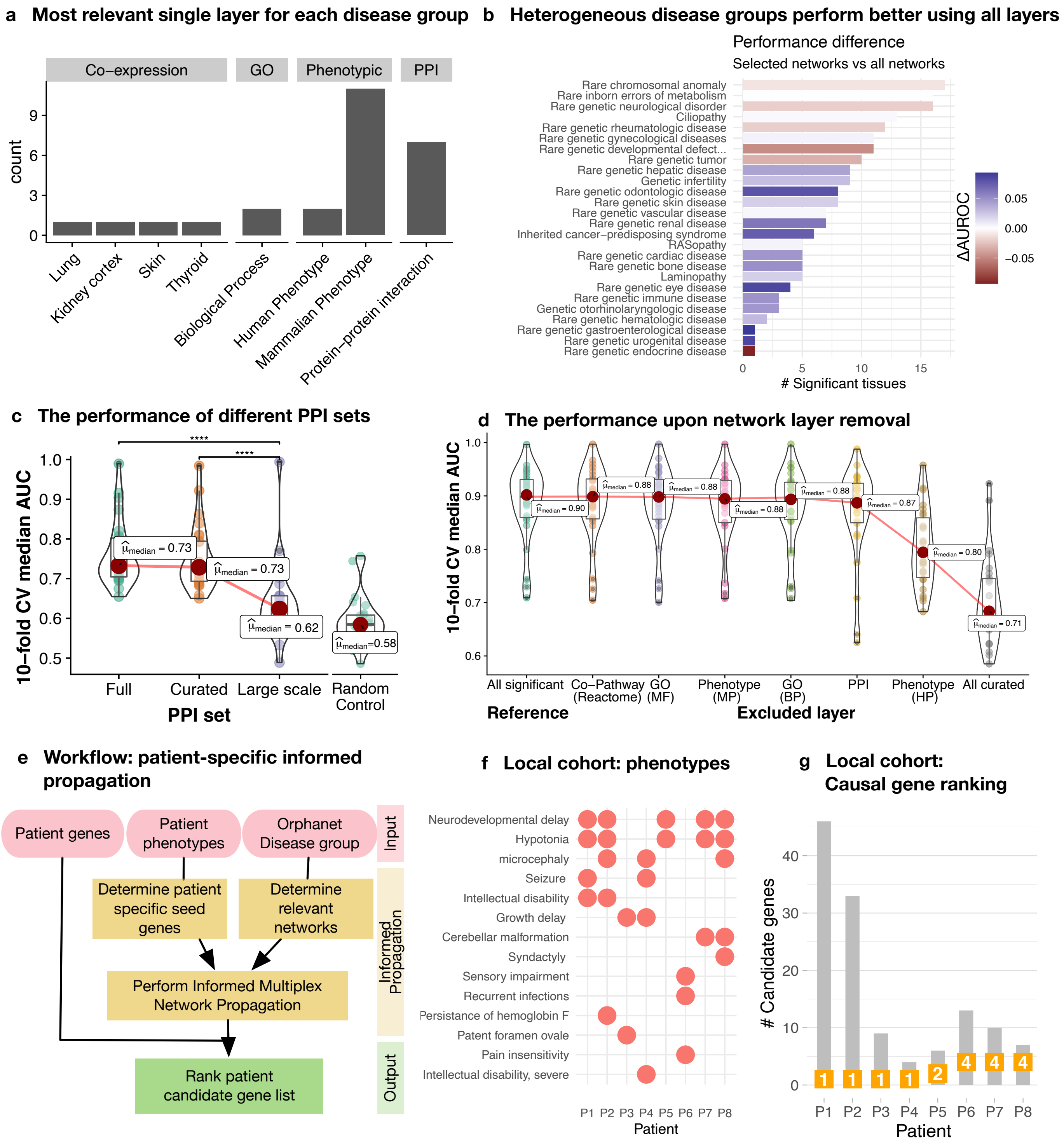

Supplementary Figure 7. Disease gene prediction and causal gene prioritization in patients. **(a)** Number of times that a particular network layer was found to be the most relevant layer (i.e., with the most significant modularity) for a particular disease. **(b)** Performance difference (ΔAUROC) between using only relevant layers and all layers. Red: All layers perform better, blue: relevant layers perform better. Inclusion of all network layers yields better retrieval performances compared to selected relevant networks in syndromic disease groups (i.e., diseases that manifest in multiple physiological systems). **(c)** 10-fold cross validation retrieval performance of disease genes for different PPI subsets (n = 26 disease groups). The curated PPI performs equally well as the full PPI (median AUROC = 0.73), whereas the unbiased PPI sees a significant performance drop (median AUROC = 0.62, p-value = 1.76e-9, FDR-corrected Durbin-Conover non-parametric test). Random subnetworks of curated PPI that have the same number of edges as the curated PPI show a comparable performance drop (with a median AUROC of 0.58, even slightly reduced). Threshold for p-values: p<0.05:*, p<0.01:**, p<0.001:***, p<0.0001:****. Bounds of box represent 25th and 75th percentiles, center the median, whiskers 10th and 90th percentiles, respectively. **(d)** Prediction performance of the multiplex network upon removing network layers derived from curated databases (n = 26 disease groups). For most layers, the 10-fold cross validation AUROC performance is only slightly decreased after their removal (median AUROC between 0.87 and 0.88 for pathway, GO, MPO and PPI layers), only the removal of the HPO layer had a stronger impact (AUROC = 0.80, p-value = 0.0003, FDR-corrected Durbin-Conover non-parametric test). Removal of all layers that involve curated data (Reactome, GO, HP, MP, and PPI) resulted in the lowest performance (AUROC = 0.71, p-value = 1.17e-11). Elements of boxplots are as described in (c). **(e)** Schematic of the patient-specific informed propagation framework for prioritizing patients' causal genes. **(f)** Phenotypic terms (HPO) associated with different patients in the local cohort. **(g)** Causal gene ranking for the local cohort (8 patients). Grey bars represent the number of candidate genes in each patient (mean = 16), yellow boxes indicate the ranking of the actual causal genes provided by our framework.

Supplementary Figure 8

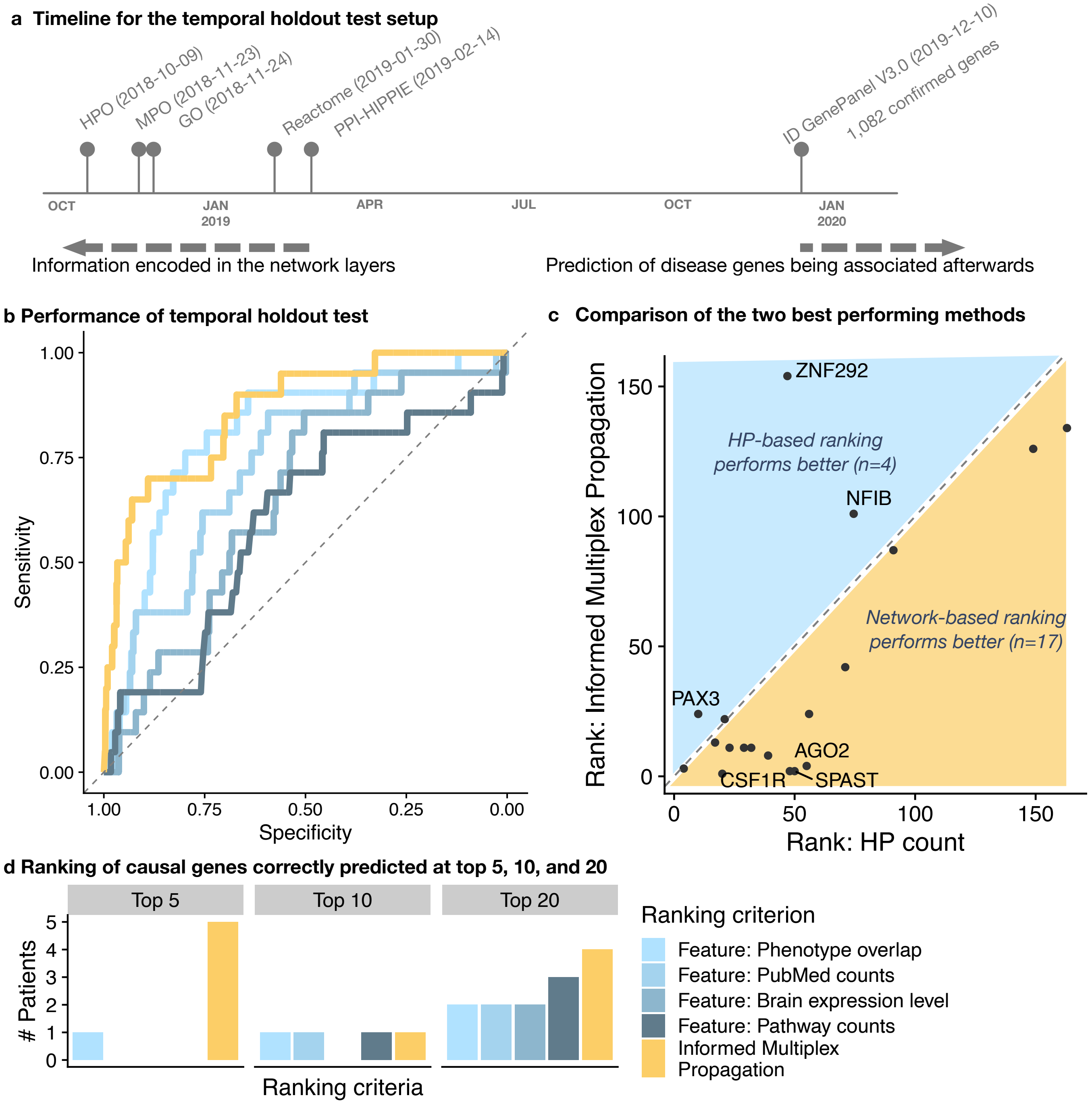

Supplementary Figure 8. Temporal-holdout analysis of causal genes discovered after network construction. (a) Timeline for identifying patients with causal genes that were discovered after the curated databases (GO, MPO, HPO, and the PPI) were retrieved. **(b)** ROC curve of our approach (yellow, AUROC = 0.95) and various gene level based benchmarks for the temporal-holdout set of 21 patients with causal genes discovered after data curation. The overall performance as measured by the AUROC remained high for all tested prediction methods, slight reductions (e.g. from 0.90 to 0.86 for the informed multiplex propagation) were within the 10-fold interquartile range in most cases **(c)** Comparison between the predicted ranks of true genes according to the two best performing methods, the informed multiplex propagation, and the phenotypic overlap. The former yields better ranking in 17 cases (81%). **(d)** Number of patients for which the true causal gene was prioritized among the top five, 10, and 20 for all considered methods.
