# Supplementary material for: Network analysis reveals rare disease signatures across multiple levels of biological organization
Source: Nat Commun. 2021 Nov 9;12:6306. doi: 10.1038/s41467-021-26674-1 (PMC8578255; doi:10.1038/s41467-021-26674-1)
Supplement: Supplementary file 2 — Reporting Summary [file 41467_2021_26674_MOESM2_ESM.pdf]

nature portfolio

Corresponding author(s): Jörg Menche

Last updated by author(s): Oct 14, 2021

Reporting Summary

Nature Portfolio wishes to improve the reproducibility of the work that we publish. This form provides structure for consistency and transparency in reporting. For further information on Nature Portfolio policies, see our Editorial Policies and the Editorial Policy Checklist.

Statistics

For all statistical analyses, confirm that the following items are present in the figure legend, table legend, main text, or Methods section.

n/a	Confirmed	
☐	☒	The exact sample size (n) for each experimental group/condition, given as a discrete number and unit of measurement
☒	☐	A statement on whether measurements were taken from distinct samples or whether the same sample was measured repeatedly
☐	☒	The statistical test(s) used AND whether they are one- or two-sided<br>*Only common tests should be described solely by name; describe more complex techniques in the Methods section.*
☒	☐	A description of all covariates tested
☐	☒	A description of any assumptions or corrections, such as tests of normality and adjustment for multiple comparisons
☒	☐	A full description of the statistical parameters including central tendency (e.g. means) or other basic estimates (e.g. regression coefficient) AND variation (e.g. standard deviation) or associated estimates of uncertainty (e.g. confidence intervals)
☐	☒	For null hypothesis testing, the test statistic (e.g. F, t, r) with confidence intervals, effect sizes, degrees of freedom and P value noted<br>*Give P values as exact values whenever suitable.*
☒	☐	For Bayesian analysis, information on the choice of priors and Markov chain Monte Carlo settings
☒	☐	For hierarchical and complex designs, identification of the appropriate level for tests and full reporting of outcomes
☐	☒	Estimates of effect sizes (e.g. Cohen's d, Pearson's r), indicating how they were calculated

Our web collection on statistics for biologists contains articles on many of the points above.

Software and code

Policy information about availability of computer code

Data collection	Data for network construction were collected using resources listed in Table 1. No additional softwares were used in the collection process.
Data analysis	Analyses were conducted using the R Statistical language (version 3.6.3; R Core Team, 2020) on macOS 10.16, using the packages voronoiTreemap (version 0.2.1; Alexander Kowarik et al., 2021), cowplot (version 1.1.1; Claus Wilke, 2020), igraph (version 1.2.6; Csardi G, Nepusz T: The igraph software package for complex network research, InterJournal, Complex Systems 1695. 2006. https://igraph.org), RColorBrewer (version 1.1.2; Erich Neuwirth, 2014), ggplot2 (version 3.3.3; Wickham. ggplot2: Elegant Graphics for Data Analysis. Springer-Verlag New York, 2016.), stringr (version 1.4.0; Hadley Wickham, 2019), tidyr (version 1.1.2; Hadley Wickham, 2020), forcats (version 0.5.1; Hadley Wickham, 2021), scales (version 1.1.1; Hadley Wickham and Dana Seidel, 2020), readr (version 1.4.0; Hadley Wickham and Jim Hester, 2020), dplyr (version 1.0.4; Hadley Wickham et al., 2021), ggforestplot (version 0.1.0; Ilari Scheinin et al., 2021), rmarkdown (version 2.7; JJ Allaire and Yihui Xie and Jonathan McPherson and Javier Luraschi and Kevin Ushey and Aron Atkins and Hadley Wickham and Joe Cheng and Winston Chang and Richard Iannone, 2021), ggrepel (version 0.9.1; Kamil Slowikowski, 2021), tibble (version 3.1.0; Kirill Müller and Hadley Wickham, 2021), purrr (version 0.3.4; Lionel Henry and Hadley Wickham, 2020), report (version 0.3.0; Makowski et al., 2020), treemap (version 2.4.2; Martijn Tennekes, 2017), ggstatsplot (version 0.7.0; Patil, 2018), pacman (version 0.5.1; Rinker et al., 2017), ggraph (version 2.0.4; Thomas Lin Pedersen, 2020), patchwork (version 1.1.1; Thomas Lin Pedersen, 2020), tidygraph (version 1.2.0; Thomas Lin Pedersen, 2020), MASS (version 7.3.53; Venables et al., 2002), tidyverse (version 1.3.0; Wickham et al., 2019), pROC (version 1.17.0.1; Xavier Robin et al., 2011) and knitr (version 1.31; Yihui Xie, 2021). Full reference list of software used can be found at https://github.com/menchelab/MultiOme/blob/main/report/report_session.md. Source code and cache data is available at the https://github.com/menchelab/MultiOme (DOI:10.5281/zenodo.5562924). The supplementary Explorer app for detailed inspection of disease-network specificity is available at www.menchelab.com/MultiOmeExplorer

For manuscripts utilizing custom algorithms or software that are central to the research but not yet described in published literature, software must be made available to editors and reviewers. We strongly encourage code deposition in a community repository (e.g. GitHub). See the Nature Portfolio guidelines for submitting code & software for further information.

Data

Policy information about availability of data

All manuscripts must include a data availability statement. This statement should provide the following information, where applicable:

- Accession codes, unique identifiers, or web links for publicly available datasets
- A description of any restrictions on data availability
- For clinical datasets or third party data, please ensure that the statement adheres to our policy

Data generated in this study are provided in the Supplementary Information/Source Data file. The RDconnect Genome-Phenome Analysis Platform (GPAP) data are available under restricted access, which can be obtained by validated users via the platform at https://platform.rd-connect.eu/.

Field-specific reporting

Please select the one below that is the best fit for your research. If you are not sure, read the appropriate sections before making your selection.

[x] Life sciences [ ] Behavioural & social sciences [ ] Ecological, evolutionary & environmental sciences

For a reference copy of the document with all sections, see nature.com/documents/nr-reporting-summary-flat.pdf

Life sciences study design

All studies must disclose on these points even when the disclosure is negative.

Sample size	For statistical analyses of network and disease characterization, sample sizes are provided in figure legends and in the Methods. For patient data, we include all patients with known causal genes for both cohorts in consideration.
Data exclusions	No data were excluded in the study.
Replication	Study does not involve experimental results that require replication. Since the study uses biological samples of patients with unique clinical phenotype and genetic profile, biological replication is not possible and not relevant.
Randomization	Randomization is not relevant to the study as there was no group allocation involved.
Blinding	Blinding is not relevant to the study as there was no group allocation involved. Genome analyses are ascertained to be blind.

Reporting for specific materials, systems and methods

We require information from authors about some types of materials, experimental systems and methods used in many studies. Here, indicate whether each material, system or method listed is relevant to your study. If you are not sure if a list item applies to your research, read the appropriate section before selecting a response.

Materials & experimental systems

n/a	Involved in the study	
x		Antibodies
x		Eukaryotic cell lines
x		Palaeontology and archaeology
x		Animals and other organisms
	x	Human research participants
x		Clinical data
x		Dual use research of concern

Methods

n/a	Involved in the study	
x		ChIP-seq
x		Flow cytometry
x		MRI-based neuroimaging

Human research participants

Policy information about studies involving human research participants

Population characteristics	Eight participants that were diagnosed with unknown rare neurological disorder of likely genetic origin were recruited to the study for genetic testing after clinical assessment.
Recruitment	Recruitment was based on the referral by the clinician, with the purpose of genetic testing.
Ethics oversight	Ethics Committee of the Medical University of Vienna; and/or Haunerschen Kinderspital, Munich, Germany; Servicio di Consulenza Genetica, Bolzano, Italy; University Hospital Zagreb, Zagreb, Croatia; General Hospital Varazdin, Varazdin, Croatia; and Tehran University of Medical Sciences, Tehran, Iran in accordance with the Declaration of Helsinki.

Note that full information on the approval of the study protocol must also be provided in the manuscript.
