# Supplementary material for: Network analysis reveals rare disease signatures across multiple levels of biological organization
Source: Nat Commun. 2021 Nov 9;12:6306. doi: 10.1038/s41467-021-26674-1 (PMC8578255; doi:10.1038/s41467-021-26674-1)
Supplement: Supplementary file 3 — Description of Additional Supplementary Files [file 41467_2021_26674_MOESM3_ESM.pdf]

Title: Supplementary Data 1:
Description: A tab-delimited file containing network details, layer representation and resources

Title: Supplementary Data 2:
Description: A zip file containing edge lists of all networks used in the analyses

Title: Supplementary Data 3:
Description: A csv file containing details of networks derived from GTEx including original tissues, merged tissue groups, and abbreviation

Title: Supplementary Data 4:
Description: A tab-delimited file containing individual orphanet disease-gene association and top-level disease group

Title: Supplementary Data 5:
Description: A tab-delimited file containing orphanet top-level disease groups and gene associations

Title: Supplementary Data 6:
Description: A csv file containing processed gene list from patients in the local cohort

Title: Supplementary Data 7:
Description: A csv file containing associated phenotypes of patients in the local cohort

Title: Supplementary Data 8:
Description: A csv file containing counts of PubMed articles associated with each gene

Title: Supplementary Data 9:
Description: A tab-delimited file containing a list of essential genes in human based on OGEE database

Title: Supplementary Data 10:
Description: A tsv file containing standard gene symbols with Ensembl identifiers used throughout the analyses

Title: Supplementary Data 11
Description: Summary of genotypes (filtered gene lists, see Methods) and phenotypes including associated H PO IDs, sex (M=male, F=female), and age (in years) of patients in the local cohort. Computer-readable formats of this dataset is provided in Supplementary Data 6 and 7.
