# Supplementary material for: Network analysis reveals rare disease signatures across multiple levels of biological organization
Source: Nat Commun. 2021 Nov 9;12:6306. doi: 10.1038/s41467-021-26674-1 (PMC8578255; doi:10.1038/s41467-021-26674-1)
Supplement: Supplementary file 14 — Supplementary Data 11 [file 41467_2021_26674_MOESM14_ESM.docx]

## Supplementary Data 11

| ID | Filtered gene list | Associated HPO IDs |
| --- | --- | --- |
| P1 | ABCA6;ABCC3;ABLIM2;ACE;ACTN3;ARFGEF2;BCOR;BRWD3;C16orf13;CABIN1;CASP8;CCDC19;CCM2;CEP290;COL27A1;EFCAB6;FAM189B;FAM208B;GLA;GRAMD4;HEATR5B;INO80;KANSL1L;KCNK7;LRRC37A3;MAGEB1;MSLN;PLA2G4D;PLA2G6;POF1B;POLA2;PRDM12;PROKR2;PSMC6;RBMXL3;RP5-1165K10.2;S100A13; SLC27A3;SLC9A3R2;SRRM2;TMEM114;TMEM114;TMEM8A;UNC80;UPK3A;XKRX;ZNF618 | HP:0007021;HP:0002719;HP:0000729;HP:0003474;HP:0010829;HP:0012804 |
| P2 | APOB;CEP41;CLN8;CNNM3;COL4A1;COL4A2;CPAMD8;CTNS;DCHS1;DPAGT1;FBN2;FRMPD3;GRIN2D;IFT140;INTS1;LAMA1;LRBA;MAPRE2;MAZ;MCM6;MYO5B;NUP107;NUP133;POLD1;PRSS56;PYCR2;SHANK1;TMEM231;TRPS1;TTN;TYMP;VIPAS39;WDR11 | HP:0001252;HP:0012758; HP:0002438 |
| P3 | ABCB7;CCDC22;FAAH2;GPR119;HUWE1; JADE3;SULT1A1;TAS2R43;ZNF414 | HP:0001510;HP:0001999;HP:0000348;HP:0001276;HP:0001655 |
| P4 | ATXN1;CNTNAP2;GOLGA6D;HIST1H2AD | HP:0001510;HP:0000252;HP:0010864;HP:0001250 |
| P5 | CDKL5;EGF;ESPN;SHH;TSC2;TYW3 | HP:0001252;HP:0001249;HP:0001250;HP:0012758 |
| P6 | ABCD1;CCDC22;CFTR;EBNA1BP2;FLNB; IGDCC4;LAMA1;MYEOV;NSD1;PYGM;RAD54L;SNX10;THEMIS2 | HP:0001252;HP:0012758; HP:0002438;HP:0001159;HP:0000252;HP:0000028 |
| P7 | ARFGEF2;BCL11A;CACNA1C;CMPK2;FREM2;MUC4;NDST1;PAFAH1B1;RUSC2;TBCK | HP:0012758;HP:0001251;HP:0011904;HP:0000252;HP:0001252;HP:0001249 |
| P8 | ARID1B;CACNA2D1;CHD2;LUZP4;NOTCH3; PLXNB3;TWNK | HP:0012758;HP:0001252;HP:0012433;HP:0002353 |

**Supplementary Data 11**: Summary of genotypes (filtered gene lists, see Methods) and phenotypes including associated HPO IDs of patients in the local cohort. Computer-readable formats of this dataset is provided in Supplementary Data 6 and 7.
